# Divergent trajectories of antiviral memory after SARS-CoV-2 infection

**DOI:** 10.1038/s41467-022-28898-1

**Published:** 2022-03-10

**Authors:** Adriana Tomic, Donal T. Skelly, Ane Ogbe, Daniel O’Connor, Matthew Pace, Emily Adland, Frances Alexander, Mohammad Ali, Kirk Allott, M. Azim Ansari, Sandra Belij-Rammerstorfer, Sagida Bibi, Luke Blackwell, Anthony Brown, Helen Brown, Breeze Cavell, Elizabeth A. Clutterbuck, Thushan de Silva, David Eyre, Sheila Lumley, Amy Flaxman, James Grist, Carl-Philipp Hackstein, Rachel Halkerston, Adam C. Harding, Jennifer Hill, Tim James, Cecilia Jay, Síle A. Johnson, Barbara Kronsteiner, Yolanda Lie, Aline Linder, Stephanie Longet, Spyridoula Marinou, Philippa C. Matthews, Jack Mellors, Christos Petropoulos, Patpong Rongkard, Cynthia Sedik, Laura Silva-Reyes, Holly Smith, Lisa Stockdale, Stephen Taylor, Stephen Thomas, Timothy Tipoe, Lance Turtle, Vinicius Adriano Vieira, Terri Wrin, OPTIC Clinical Group, Lizzie Stafford, Hibatullah Abuelgasim, Ahmed Alhussni, Carolina V. Arancibia-Cárcamo, Martyna Borak, Joseph Cutteridge, Alexandra Deeks, Lucy Denly, Stavros Dimitriadis, Shayan Fassih, Thomas Foord, Thomas Fordwoh, Jennifer Holmes, Bryn Horsington, Sven Kerneis, David Kim, Katy Lillie, Jordan Morrow, Denise O’Donnell, Thomas G. Ritter, Beatrice Simmons, Adan Taylor, Sarah R. Thomas, Yolanda Warren, Adam J. R. Watson, Esme Weeks, Robert Wilson, Rebecca Young, PITCH Study Group, Christopher J. A. Duncan, Shona C. Moore, Rebecca Payne, Alex Richter, Sarah Rowland-Jones, C-MORE Group, Alexander J. Mentzer, Mark Philip Cassar, Tao Dong, Anastasia Fries, Javier Gilbert-Jaramillo, Ling-Pei Ho, Julian C. Knight, Stefan Neubauer, Yanchun Peng, Nayia Petousi, Betty Raman, Nick P. Talbot, Andrew J. Pollard, Teresa Lambe, Chris P. Conlon, Katie Jeffery, Simon Travis, Philip Goulder, John Frater, Alex J. Mentzer, Lizzie Stafford, Miles W. Carroll, William S. James, Paul Klenerman, Eleanor Barnes, Christina Dold, Susanna J. Dunachie

**Affiliations:** 1 Oxford Vaccine Group, Department of Paediatrics, University of Oxford, Oxford, UK; 2 Peter Medawar Building for Pathogen Research, Nuffield Dept. of Clinical Medicine, University of Oxford, Oxford, UK; 3 Oxford University Hospitals NHS Foundation Trust, Oxford, UK; 4 Nuffield Dept of Clinical Neuroscience, University of Oxford, Oxford, UK; 5 NIHR Oxford Biomedical Research Centre, Oxford, UK; 6 United Kingdom Health Security Agency, Porton Down, Wiltshire, England; 7 Department of Clinical Biochemistry, Oxford University Hospitals NHS Foundation Trust, Oxford, UK; 8 Jenner Institute, University of Oxford, Oxford, UK; 9 The Florey Institute for Host-Pathogen Interactions and Department of Infection, Immunity and Cardiovascular Disease, Medical School, University of Sheffield, Sheffield, UK; 10 Big Data Institute, Nuffield Dept. of Population Health, University of Oxford, Oxford, UK; 11 Department of Physiology, Anatomy, and Genetics, University of Oxford, Oxford, UK; 12 James & Lillian Martin Centre, Sir William Dunn School of Pathology, University of Oxford, Oxford, UK; 13 Oxford University Medical School, Medical Sciences Division, University of Oxford, Oxford, UK; 14 Oxford Centre For Global Health Research, Nuffield Dept. of Clinical Medicine, University of Oxford, Oxford, UK; 15 Monogram Biosciences LabCorp, San Francisco, CA USA; 16 Wellcome Centre for Human Genetics, University of Oxford, Oxford, UK; 17 Mahidol-Oxford Tropical Medicine Research Unit, Mahidol University, Bangkok, Thailand; 18 HPRU in Emerging and Zoonotic Infections, Institute of Infection, Veterinary and Ecological Sciences, University of Liverpool, Liverpool, UK; 19 Tropical and Infectious Disease Unit, Liverpool University Hospitals NHS Foundation Trust (a member of Liverpool Health Partners), Liverpool, UK; 20 Peter Medawar Building for Pathogen Research, Department of Paediatrics, University of Oxford, Oxford, UK; 21 Nuffield Department of Medicine, University of Oxford, Oxford, UK; 22 Radcliffe Department of Medicine, University of Oxford, Oxford, UK; 23 Translational Gastroenterology Unit, Nuffield Department of Medicine, University of Oxford, Oxford, UK; 24 Oxford University Medical School, University of Oxford, Oxford, UK; 25 Department of Infection and Tropical Medicine, Newcastle upon Tyne Hospitals NHS Foundation, Newcastle upon Tyne, UK; 26 Translational and Clinical Research Institute, Department of Infection and Tropical Medicine, Newcastle upon Tyne Hospitals NHS Foundation, Newcastle upon Tyne, UK; 27 HPRU in Emerging and Zoonotic Infections, Institute of Infection, Veterinary and Ecological Sciences, University of Liverpool, Liverpool, UK; 28 Translational and Clinical Research Institute Immunity and Inflammation Theme, Newcastle University, Newcastle, UK; 29 Institute of Immunology and Immunotherapy, College of Medical and Dental Science, University of Birmingham, Birmingham, UK; 30 University Hospitals Birmingham NHS Foundation Trust, Birmingham, UK; 31 Sheffield Teaching Hospitals NHS Foundation Trust, Sheffield, UK; 32 Department of Infection, Immunity and Cardiovascular Disease, University of Sheffield, Sheffield, UK; 33 MRC Human Immunology Unit, MRC Weatherall Institute of Molecular Medicine, University of Oxford, Oxford, UK; 34 Chinese Academy of Medical Science Oxford Institute (COI), University of Oxford, Oxford, UK; 35 Department of Physiology, Anatomy and Genetics, University of Oxford, Oxford, UK

**Keywords:** Viral infection, Infection, SARS-CoV-2, Immunological memory

## Abstract

The trajectories of acquired immunity to severe acute respiratory syndrome coronavirus 2 infection are not fully understood. We present a detailed longitudinal cohort study of UK healthcare workers prior to vaccination, presenting April-June 2020 with asymptomatic or symptomatic infection. Here we show a highly variable range of responses, some of which (T cell interferon-gamma ELISpot, N-specific antibody) wane over time, while others (spike-specific antibody, B cell memory ELISpot) are stable. We use integrative analysis and a machine-learning approach (SIMON - Sequential Iterative Modeling OverNight) to explore this heterogeneity. We identify a subgroup of participants with higher antibody responses and interferon-gamma ELISpot T cell responses, and a robust trajectory for longer term immunity associates with higher levels of neutralising antibodies against the infecting (Victoria) strain and also against variants B.1.1.7 (alpha) and B.1.351 (beta). These variable trajectories following early priming may define subsequent protection from severe disease from novel variants.

## Abstract (web summary)

The engagement of immunological memory is a key component to the protective anti-SARS-CoV-2 B and T cell responses. Here the authors assess the B and T cells of a cohort of UK healthcare workers in response to infection and longitudinally track the compartment showing distinct trajectories following early priming.

## Introduction

Severe acute respiratory syndrome coronavirus 2 (SARS-CoV-2), an RNA virus that causes coronavirus disease 2019 (COVID-19), first emerged in humans in December 2019 and has since spread globally, with more than 3.56 million deaths reported world-wide (June 2021 https://coronavirus.jhu.edu/map.html). Although the majority of infections cause asymptomatic or mild disease, a significant minority develop a severe illness, requiring hospitalisation, oxygen support, and invasive ventilation^1^. Healthcare workers (HCW) have been at the forefront of caring for patients with SARS-CoV-2 infection in community and hospital environments during the pandemic. High exposure rates have meant that a significant proportion of HCW have become infected and HCW most commonly infected are those working on the front line in patient facing roles, predominantly in acute medical specialities^2^. Older age, comorbidities and male sex remain the dominant factors that predispose to severe outcomes^3^—since HCW are predominantly younger and female^2^, most have developed mild disease, although deaths are widely reported in this population.

Starting early in the pandemic, we and others have sought to characterise the immune responses during SARS-CoV-2 infection that are associated with viral clearance and disease severity. SARS-CoV-2 infection has been associated with the generation of high magnitude, broad T cell responses and high titres of immunoglobulin G (IgG) targeting SARS-CoV-2 spike and nucleoprotein (NP) antigens, particularly in severe COVID-19^4^. Asymptomatic infection, that appears more common in younger people, may be associated with discordant T cell and humoral immunity with both the absence of IgG seroconversion in the presence of detectable T cell responses^5,6^ or conversely the presence of IgG in the absence of T cell immune responses^7^. However, more recently critical questions have emerged that include the durability of immune responses following initial infection, the quality of these responses, immune correlates of protection from re-infection, and the capacity of these responses to neutralise new variants of concern (VOC) that have emerged globally. These questions have become paramount following the development of effective vaccines for COVID-19, since deployment of these has been limited by vaccine supply, concerns around adverse events and vaccine hesitancy. Furthermore, to manage limited vaccine resource, people with previous infection are now being offered a single vaccine dose 6 months after infection in many European countries (France, Germany, Spain, and Italy)^8^, on the assumption that past immunity will protect from re-infection.

An in depth understanding of immune responses after SARS-CoV-2 infection, and how these change over time, will be critical to understanding who is susceptible to re-infection and to inform vaccine strategies. Currently, the precise correlates of immune protection from subsequent infection after primary disease, or after vaccination, are unknown. Previous reports suggest SARS-CoV-2 IgG antibodies^9^ and previous exposure to seasonal coronaviruses (CoV)^10^ are protective against subsequent SARS-CoV-2 infection. However, since the magnitude of T and B cell responses correlate with each other^11^, dissecting the role of these immune subsets in protection from re-infection or severe disease on re-exposure is challenging. Several groups have now reported that SARS-CoV-2 specific T and B cells decline after acute disease^12–16^, but there is high heterogeneity between individuals in the levels of measurable immunity in different compartments it is unclear how or if the kinetics of this decline correlate with protection from subsequent infection. Concerns have been raised that SARS-CoV-2 re-infection associated with waning immunity is plausible, particularly since the seasonal coronaviruses, closely related to SARS-CoV-2, commonly re-infect the same host^17–19^. However, waning of immune responses following acute infection, or vaccination is well recognised as part of the normal evolution of memory responses, and reports describing decline in immune responses have focused on ex vivo responses that may not reflect the memory recall potential of viral specific T and B cells responses. A particular concern is the identification of SARS-CoV-2 variants of concern (VOC) (B.1.1.7-alpha, B.1.351-beta, P.1-gamma and B.1.617.2-delta), with mutations which are associated with an increase in transmissibility, severity or escape from vaccine or SARS-CoV-2-induced immunity^20–28^. Reduced neutralisation of VOC, in live viral assays in vitro, appears following vaccination and after SARS-CoV-2 infection, and is pronounced in the context of lower antibody titres measured against the initial pandemic strain (B/Victoria)^21,23,26,29^.

Since April 2020, we have followed a cohort of SARS-CoV-2 infected HCW prospectively over time at Oxford University Hospital NHS Foundation Trust. Seventy-eight HCW infected during the UK’s ‘first wave’ (defined by positive PCR and seropositive for anti-spike antibodies) were assessed at up to six timepoints and followed for six months in 2020, pre-vaccination, with multiple immune parameters evaluated in more than 430 blood draws. Our aims are to characterise memory T and B cell responses following infection, and to determine the interactions between clinical presentation and the generation and maintenance of T and B cell responses over time. We assess the association of exposure to seasonal coronaviruses and symptomatic SARS-CoV-2 disease with the durability of SARS-CoV-2 specific responses. We evaluate the predictive value of clinical and immune parameters measured early after infection on the durability of immune responses using an integrative analysis with a machine learning platform (SIMON)^30,31^. Using this approach, we define a group of high and low antibody responders with a differential capacity to neutralise the VOC.

## Results

### Anti-spike IgG and memory responses are maintained, whilst anti-nucleocapsid IgG decline over time and stratify by disease severity

**Fig. 1:**
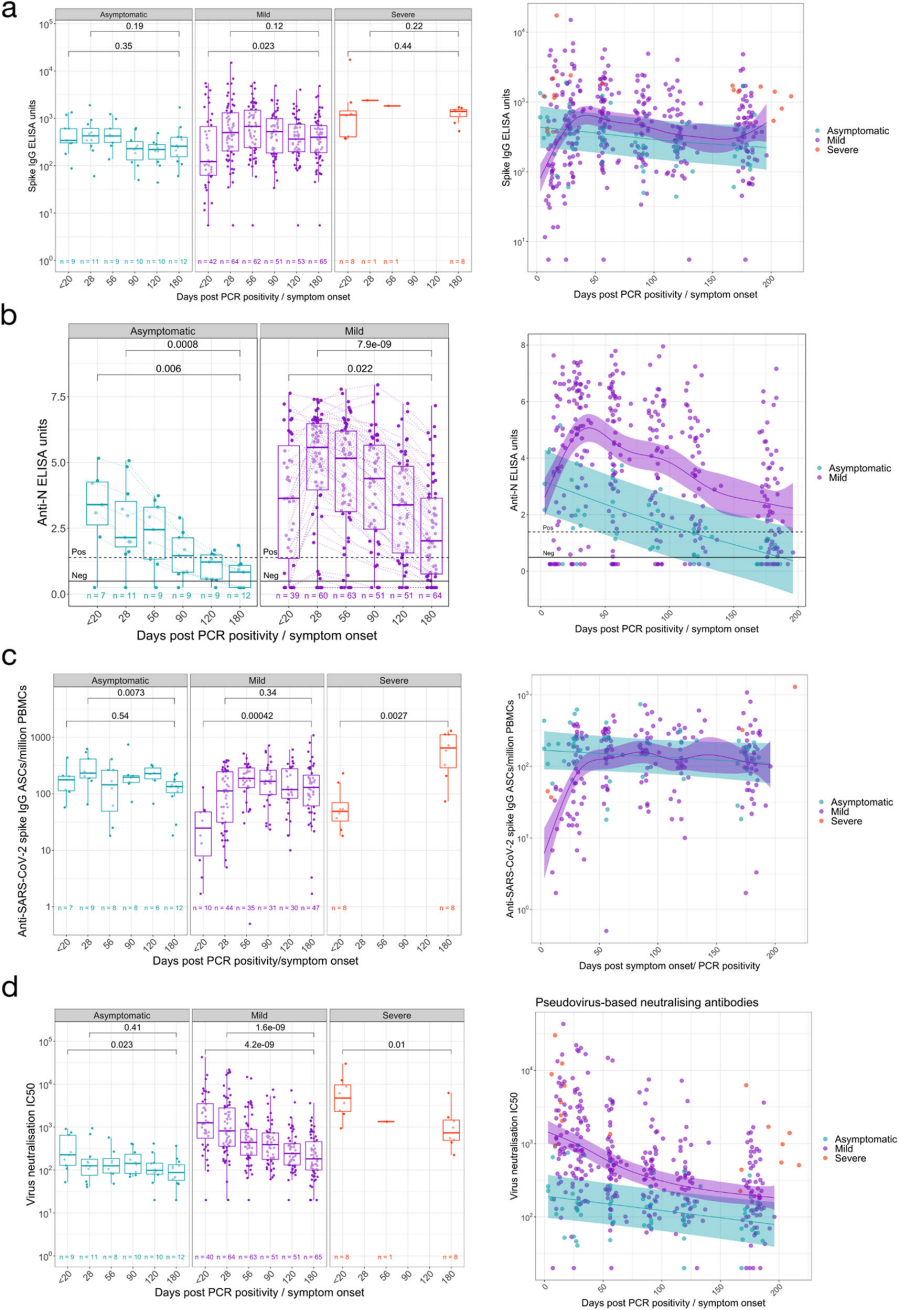
Longitudinal humoral immune responses in individuals with PCR confirmed SARS-CoV-2 asymptomatic, mild or severe infection. Humoral immune responses were assessed in acute and convalescent by binding antibody ELISA for total IgG specific to the **a** Spike glycoprotein and **b** Nucleocapsid, quantification of **c** IgG memory B cells specific to the spike glycoprotein, and **d** pseudoneutralisation antibody titres. A two-tailed Wilcoxon rank-sum test was used to compare between study time points. The boxplots all display the median values with the first and third quartile, and the whiskers represent the highest and lowest values no more than 1.5 times the interquartile range from the corresponding hinge. A generalised additive mixed model (GAMM) by restricted maximum likelihood—right-hand plots—was used to fit the immunological measures (log10 transformed) taken at multiple study time points, using Gaussian process smooth term. The GAMM plots the ribbon represents the 95% confidence interval around the fitted value. Disease severity group was included in the GAMM as a linear predictor and a participant identifier was included as a random effect. See Table S1 for number of individuals evaluated per assay.

Anti-spike (S) and nucleocapsid (NP) total IgG (tIgG) responses were assessed by ELISA in both symptomatic and asymptomatic individuals (Fig. 1a and b). The magnitude of the IgG response varied markedly between people in both cohorts, with a proportion of individuals’ anti-nucleocapsid tIgG level recorded in the negative or indeterminate range of the assay at all time-points.

Over the time course of observation, anti-spike IgG antibody levels (Fig. 1a) in individuals remained consistent in individuals with asymptomatic (*P* = 0.35) and severe *(P* = 0.44) COVID-19 disease. Similarly, the initial anti-spike tIgG responses increased in individuals with mild disease and remained consistent from day 28 to the 6-month timepoint *(P* = 0.12). Furthermore, disease severity was not a significant predictor of anti-spike tIgG levels in those with asymptomatic and mild SARS-CoV-2 infection throughout the 6-month observation (*P* = 0.632, GAMM, Fig. 1a).

Asymptomatic and mild infection induces similar anti-NP responses in the early phase (<20 days post PCR positivity/symptom onset) of observed infection (*P* = 0.6125, Supplementary Fig. 1B). However, anti-NP tIgG levels in the two disease cohorts separated as higher levels were observed in those with mild infection from the day 28 timepoint onwards (*P* = 0.0015 for day 28 comparison, Supplementary Fig. 1B). Anti-NP IgG responses waned over time with a significant decrease from approximately day 28 to day 180 timepoints (*P* = 0.00071 for asymptomatic and *P* = 7.2 × 10^−9^ for mild symptomatic individuals, Fig. 1b). Most (91.7%) asymptomatic individuals have an indeterminate or negative anti-NP tIgG response to the nucleocapsid antigen at the day 180 timepoint.

In line with the tIgG antibody binding to spike remaining consistent, we observed a steady number of IgG+ memory B cells following an initial increase (Fig. 1c). Anti-SARS-CoV-2 spike-specific IgG+ memory B cells at 6 months following symptom onset were higher than observed during early infection in mild (*P* = 0.00042, Fig. 1c) and severe (*P* = 0.0027, Fig. 1c) individuals. For asymptomatic individuals, no change was observed in cell frequencies when comparing the earliest samples collected and 6-month timepoints (*P* = 0.54), although we note that the timing of infection onset for asymptomatic individuals cannot be precisely determined. Asymptomatic and mild disease did not predict different kinetics for the IgG memory response (*P* = 0.284, GAMM, Fig. 1c).

### Pseudo-neutralisating antibodies decreased in all disease severities over time

Pseudo-neutralisating antibodies (pseudoNA) were measured in all individuals (Fig. 1d) using an assay that incorporates the spike glycoprotein. Disease severity was a significant predictor of pseudoNA (*P* = 0.00073, GAMM, Fig. 1d)—with higher pseudoNA levels with increasing disease severity at all time points measured (Fig. 1d and Supplementary Fig. 1D). Regardless of disease severity, the pseudo-neutralising capacity of circulating antibodies to the Wuhan/B lineage virus decreased over 6 months following the detection of SARS-CoV-2 infection (asymptomatic *P* = 0.023; mild *P* = 4.2 × 10^−9^; severe *P* = 0.01, Fig. 1d). People with severe infection maintained pseudoNA 6 months post symptom onset, and at higher levels than in those with mild or asymptomatic infection (*P* = 0.00022, Kruskal-Wallis test, Supplementary Fig. 1D). The decline was less marked in asymptomatic individuals with no decrease observed from day 28 to day 180 (*P* = 0.41, Fig. 1d); however, the difference in the pseudoNA titres in the mild vs asymptomatic groups remained until day 180 (*P* = 0.0148). At day 180 post symptom onset or PCR confirmation, one asymptomatic and four symptomatic individuals no longer mounted a positive result in the pseudoNA assay, one of whom consistently did not mount pseudoNA capacity at all time points measured.

### Mild infection induces a more multifunctional antibody profile

A cohort of 30 individuals with mild infection, along with the 9 and 12 participants with severe and asymptomatic infection respectively were selected to comprehensively characterise antibody profiles.

### Circulating isotypes and subclasses

**Fig. 2:**
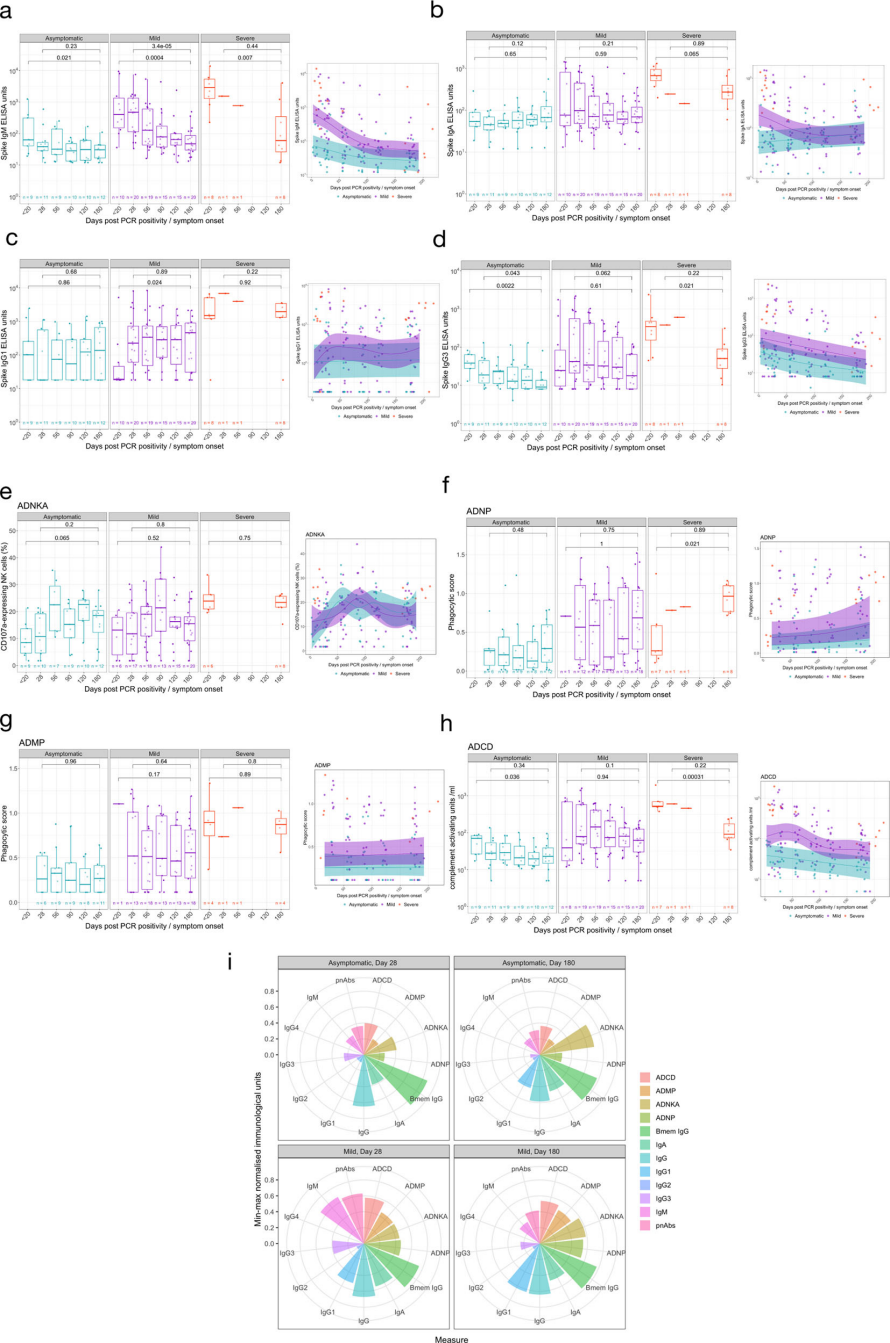
Antibody isotype, subclass and function in individuals with PCR confirmed SARS-CoV-2 asymptomatic, mild or severe infection. SARS-CoV-2 spike-specific antibody isotype and subclasses measured post-infection: **a** IgM, **b** IgA, **c** IgG1 and **d** IgG3. Antibody function measure post-SARS-CoV-2 infection: **e** antibody-dependent NK cell activation (ADNKA), **f** antibody-dependent neutrophil phagocytosis (ADNP), **g** antibody-dependent monocyte phagocytosis (ADMP) and **h** antibody-dependent complement deposition (ADCD). **i** Polar plot of various antibody isotype, subclass and function data, minimum-maximum normalised. The boxplots all display the median values with the first and third quartile, and the whiskers represent the highest and lowest values no more than 1.5 times the interquartile range from the corresponding hinge. A two-tailed Wilcoxon rank-sum test was used to compare between study time points. A generalised additive mixed model (GAMM) by restricted maximum likelihood—right-hand plots—was used to fit the immunological measures (log10 transformed) taken at multiple study time points, using Gaussian process smooth term. The GAMM plots the ribbon represents the 95% confidence interval around the fitted value. Disease severity group was included in the GAMM as a linear predictor and a participant identifier was included as a random effect. See Table S1 for number of individuals evaluated per assay.

Circulating IgM levels decreased over time in those with asymptomatic (*P* = 0.021, day <20 vs day 180), mild (*P* = 0.0004, day <20 vs day 180) and severe (*P* = 0.007, day <20 vs day 180) infection, while IgA levels in participants remained constant in all disease cohorts (asymptomatic: *P* = 0.65; mild: *P* = 0.59; severe: *P* = 0.065), throughout the observed 6-month time course (Figs. 2a, 2b) as previously reported^12^. The quantified amounts of IgG1 were consistent over time in asymptomatic (*P* = 0.86, day <20 vs day 180) and severe (*P* = 0.92, day <20 vs day 180) infection. Despite initial low titres of IgG1 in participants with mild infection, IgG1 circulating antibody titres were maintained from day 28 to 6 months post symptom onset (*P* = 0.89, Fig. 2c). While circulating IgG3 antibodies in participants with mild infection were maintained at consistent levels throughout the 6-month period (*P* = 0.062), levels decreased over this time in asymptomatic (*P* = 0.0022, day <20 vs day 180) and severe (*P* = 0.021, day <20 vs day 180) individuals (Fig. 2d). Notable SARS-CoV-2 spike-specific IgG2 responses were only detected at one or more time-points in a small number of individuals tested (asymptomatic: 3/12; mild: 3/30; severe: 1/8) (Supplementary Fig. 2B), while there was no spike-specific IgG4 detected above the LLOQ of the ELISA. For all IgG subclasses detected, asymptomatic or mild disease severity were not significant predictors of responses over time (IgG1: *P* = 0.36; IgG2: *P* = 0.92; IgG3: *P* = 0.0519, GAMM, Fig. 2c–d). All paired analysis was by Wilcoxon rank sum test.

### Diversity of antibody responses

We measured the ability of the anti-spike antibodies in those with severe or asymptomatic infection as well as a selection of individuals with mild infection, to induce innate effector functions: ADNP, ADMP, ADNKA and ADCD.

Asymptomatic and mild disease severity was not a significant predictor of Fc-mediated effector functional responses (ADNKA *P* = 0.798; ADMP *P* = 0.117; ADNP *P* = 0.206) except for ADCD (*P* = 0.00314) (Fig. 2e–h). Furthermore, normalised ADMP and ADNP scores, as well as the percentage of CD107a-expressing NK cells were stable over time, between 28 days and 180 days post symptom onset or PCR confirmation for those with asymptomatic (ADMP: *P* = 0.96; ADNP: *P* = 0.48; ADNKA: *P* = 0.2) and mild (ADMP: *P* = 0.64; ADNP: *P* = 0.75; ADNKA: *P* = 0.8) infection (Fig. 2e–h). Similarly, no decline was observed for these Fc-mediated functions from the acute sampling to 6 months post symptom onset in the severe cohort (ADMP: *P* = 0.89; ADNP: increase *P* = 0.021; ADNKA: *P* = 0.075) with the ADNP increasing over time (*P* = 0.021) (Fig. 2e–h). ADCD waned dramatically in those with severe disease over the 6-month period (*P* = 0.00031) but similarly to the other Fc-mediated functions, ADCD remained consistent from day 28 to day 180 in asymptomatic (*P* = 0.34) and mild (*P* = 0.1) infection (Fig. 2e–h). Despite waning over time, ADCD responses differed amongst the disease severity groups out until day 180 (*P* = 0.0032, Kruskal-Wallis test, Supplementary Figure 1L). All paired analysis were by Wilcoxon rank sum test.

We visualised the relative contribution of each of the anti-SARS-CoV-2 spike antibody feature in Fig. 2i. The polar plots demonstrate the diversity of asymptomatic and mild infection-induced antibody characteristics and functions on day 28 and day 180. Each wedge represents an antibody feature, and the size of each wedge is indicative of the magnitude of the response. The consistently high spike-specific IgG and spike-specific IgG+ memory B cells is clearly reflected in these plots for both mild and asymptomatic individuals. For both day 28 and day 180, a more multifunctional response was observed in individuals with mild infection, particularly for the antibody-dependent phagocytosis effector functions, which contribute markedly less to the antibody profile of asymptomatic individuals. Over time, few marked changes were observed in the relative contribution of the SARS-COV-2-specific antibody features in asymptomatic individuals, apart from an increased contribution of IgG1 and ADNKA, and decreased IgG3. Similarly, for individuals with mild infection, substantial relative decreases in IgM, pseudo-neutralising antibodies, IgA and IgG3 were noted, as well as relative increases in ADNKA and ADNP to the antibody profile.

### SARS-CoV-2 infection elicits transient cross-reactive antibodies and memory B cells specific for other circulating coronaviruses

**Fig. 3:**
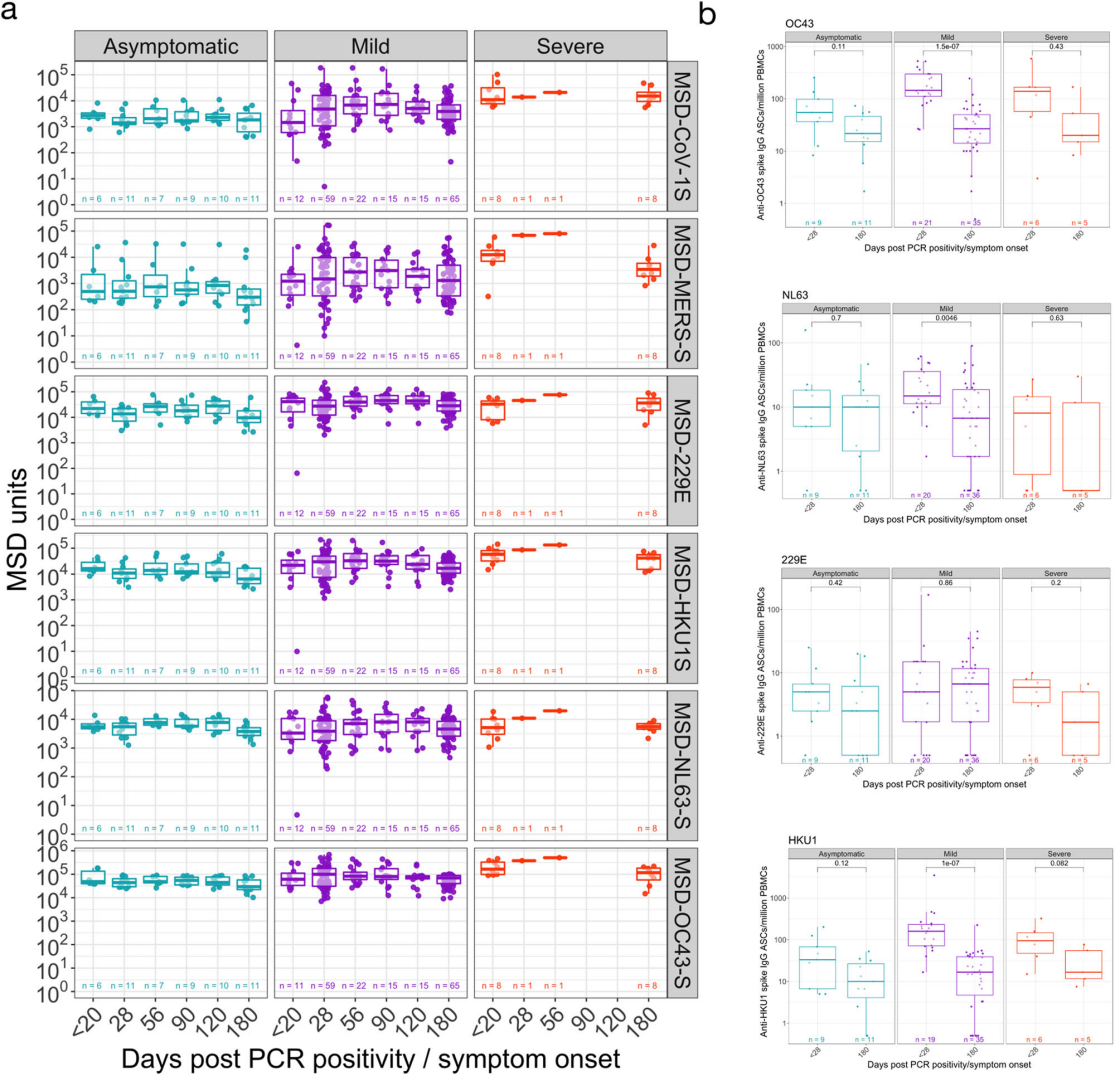
Longitudinal specific-IgG and memory B cell responses to spike protein from non-SARS-CoV-2 coronaviruses. **a** Meso Scale Discovery (MSD) multiplexed immunoassay (MIA) platform measurements of antibody levels to spike protein from non-SARS-CoV-2 coronaviruses. **b** Memory B cells responses to spike protein from non-SARS-CoV-2 coronaviruses. The boxplots all display the median values with the first and third quartile, and the whiskers represent the highest and lowest values no more than 1.5 times the interquartile range from the corresponding hinge. A two-tailed Wilcoxon rank-sum test was used to compare between study time points (without correction for multiple testing). See Table S1 for number of individuals evaluated per assay.

Next, we evaluated the IgG responses to seasonal coronaviruses (229E, HKU-1, NL63-S and OC43-S) severe acute respiratory syndrome (SARS-CoV-1) spike protein and Middle East Respiratory Syndrome (MERS) virus spike protein using the MSD assay (Fig. 3a). IgG responses to these viral antigens were detected at the earliest time points. The kinetics of these IgG responses followed those seen to SARS-CoV-2 spike, suggesting that seasonal coronavirus cross-reactive responses were enhanced by SARS-CoV-2 infection. Responses to OC43-S, 229-E and HKU-1 were particularly high and correlated significantly with disease severity at day 180 and at the earliest time point assessed (day < 20) (Supplementary Figure 2C). The MSD assay also measured IgG responses against SARS-COV-2 Spike, NP and the RBD antigens, supporting our observations using the ELISA assay (Supplementary Fig. 2D).

IgG+ Memory B cells specific for the spike glycoprotein from seasonal coronaviruses (229E, HKU1, NL63 and OC43) were determined at the earliest timepoint available (acute < day 20 or day 28) and the 6-month final sampling (Fig. 3b). The lowest responses were observed in 229E and NL63 spike IgG+ ASCs following polyclonal stimulation, which also were consistent over time with the exception of the decreased number of NL63 spike-specific IgG+ memory B cells in individuals with mild infection (*P* = 0.0046). Higher responses were detected when testing the specificity of cultured PBMCs to the beta-coronaviruses (HKU1 and OC43) spike glycoprotein. However, the boosted memory response was transient, particularly in individuals with mild infection (HKU1: *P* = 1 × 10^−7^; OC43: *P* = 1.5 × 10^−7^) in which the decrease was more marked, which may be due to a higher sample number.

### Effector poly-specific SARS-CoV-2 T cells are higher in those with mild symptoms and decline 6 months after infection

**Fig. 4:**
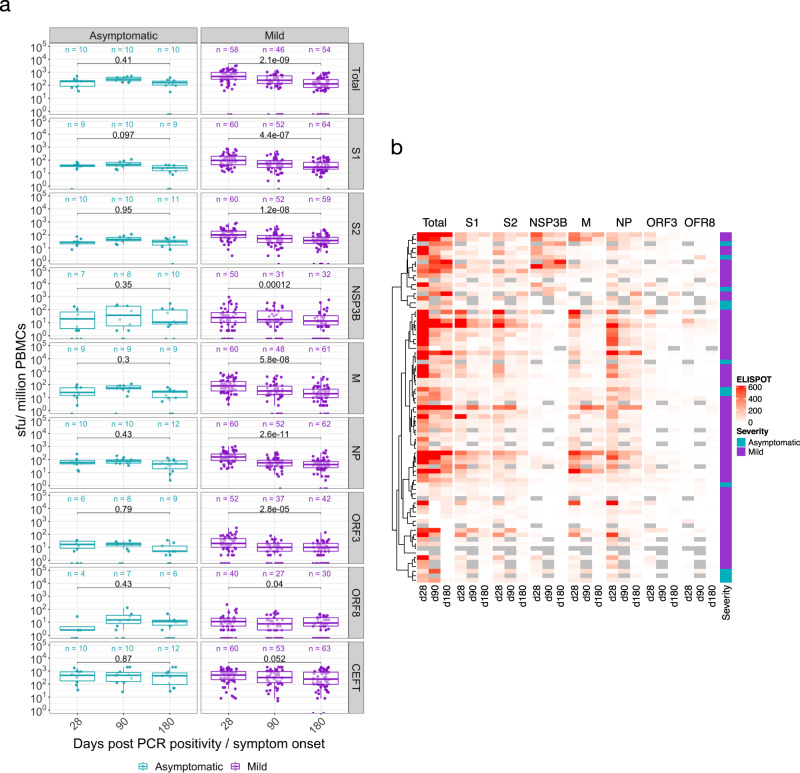
Magnitude of SARS-CoV-2 specific Effector T cell Response. **a** Ex vivo IFN-γ ELISpot showing the effector T cell responses to summed SARS-CoV-2 peptide pools spanning spike, accessory and structural proteins (summed total of SARS-CoV-2 proteins tested, S1, S2, NSP3B, M, NP, ORF 3, ORF8 and the CEFT positive control peptides for T cell responses) in 78 individuals 28, 90 and 180 days after mild or asymptomatic SARS-CoV-2 infection (onset of symptoms for mild cases, PCR positive test for asymptomatic participants). The boxplots all display the median values with the first and third quartile, and the whiskers represent the highest and lowest values no more than 1.5 times the interquartile range from the corresponding hinge. A two-tailed Wilcoxon rank-sum test was used to compare between study time points (without correction for multiple testing). **b** Heatmap displaying unsupervised hierarchical clustering of the ELISpot data in **a** and disease severity (mild or asymptomatic) for the original SARS-CoV-2 diagnosis. Sfu/million PBMCs = spot forming units per million peripheral blood mononuclear cells, with background subtracted. D28, d90 and d180 = days after SARS-CoV-2 diagnosis. Grey regions on heatmap represent missing data due to insufficient cells.

We examined the magnitude of the T cell response to SARS-CoV-2 using an ex vivo IFN-γ ELISpot assay at 28 days, 90-120 days and 180 days after SARS-CoV-2 infection *N* = 64–78 HCW/timepoint, 57 participants at all timepoints (including 12 with asymptomatic infection), and 6 volunteers with severe COVID-19 at day 180 (Figs. 4a and 4b and Supplementary Table 3). We have previously shown that this assay is specific for SARS-CoV-2, with negligible responses detected in SARS-CoV-2 pre-pandemic unexposed participants^5^.

IFN-γ responses to at least one antigenic pool were seen in 67/70 (96%) volunteers tested 28 days after SARS-CoV-2, with a median total response across the pools of 373 (IQR 201-842) SFC/10^6^ PBMC; here a response to spike (S1 and S2) was seen in 61/70 tested (87%) median 180 (IQR 71-364) SFC/10^6^ PBMC, for M in 47/70 (67%) median 63 (IQR 25-160) SFC/10^6^ PBMC and for NP in 62/70 (89%) median 121 (IQR 73-250) SFC/10^6^ PBMC. However, total summed responses declined by a median of 60% after 90 days, and by 75% at 180 days (Supplementary Table 3). The majority (61/77 (79%)) of participants had detectable responses to at least one antigenic pool at 180 days, with responses to NP antigen most commonly observed 47/77 (61%) median 40 (IQR 23-73) SFC/10^6^ PBMC. Responses to ORF3, ORF8 and NSP3B were less frequent than responses to S1, S2, M and NP at day 28 and lower at day 180.

IFN-γ ELISpot responses to SARS-CoV-2 antigens were higher in the mild symptomatic cohort (*n* = 66), compared to the asymptomatic group (*n* = 12) at 28 days, with median responses to all summed pools 455 (IQR 252-976) SFC/10^6^ PBMC for mild disease compared to 196 (IQR 74-243) SFC/10^6^ PBMC in the asymptomatic group (Supplementary Fig. 3A). There was no significant change in the magnitude of the T cell response in the asymptomatic group in the 6 months after infection (Fig. 4a).

We next used ICS to examine the duration of multiple T cell functions and the polyfunctionality of the T cell response over time at 28 and 180 days pso in individuals with ex vivo T cell ELISpot levels >100 SFC/10^6^ PBMC for sensitivity reasons (*n* = 18) with *n* = 15 available at both timepoints for paired analysis (Gating strategy in Supplementary Fig. 3D, results in Supplementary Figs. 4 and 5). Similar to the ELISpot data, the majority of T cell responses decreased over time. In terms of functionality, we found that CD4 + T cells were polyfunctional, with the majority of cells expressing >1 and up to all 5 functional markers at both timepoints. Similarly, NSP3B-specific CD8 + T cells were also polyfunctional at both timepoints examined, with most cells expressing >1 functional marker (Supplementary Fig. 4J). There were no functional changes between the two timepoints.

### T cell memory proliferative responses decline 6 months post SARS-CoV-2

We and others have found the assessment of T cell proliferation to be a sensitive method of detecting antigen-specific recall responses^5^. We used this assay to evaluate the frequency of circulating SARS-CoV-2-specific CD4+ and CD8+ T cells in our longitudinal cohort (*n* = 54–57; gating strategy presented in Supplementary Figure 3B).

**Fig. 5:**
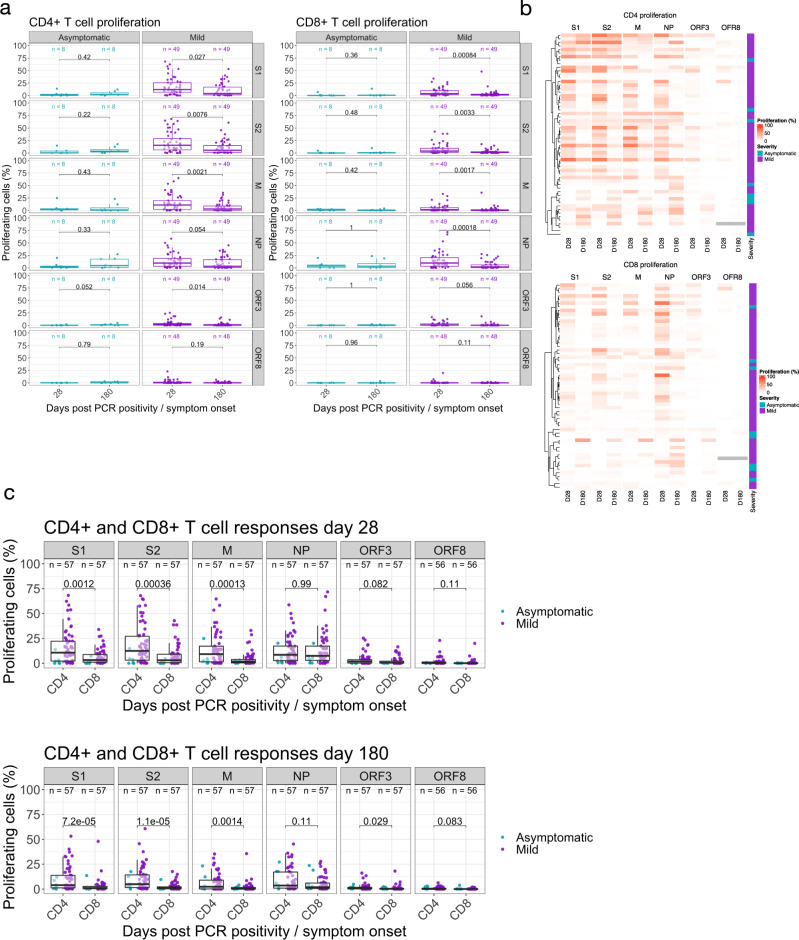
Proliferative responses to SARS-CoV-2 peptide pools at 1- and 6-months post infection. Proliferative responses against **a** SARS-CoV-2 proteins S1, S2, M, NP, ORF3 and ORF8 presented in CD4+ (Left hand panel) and CD8+ (Right hand panel) T cells measured at 28 and 180 days pso for volunteers with mild disease or days post PCR positivity for asymptomatic disease (asymptomatic *n* = 8, mild disease *n* = 49). A two-tailed Wilcoxon rank-sum test was used to compare between study time points and *P* values are indicated. **b** Unsupervised hierarchical clustering showing visual representation of SARS-CoV-2 specific responses at day 28 and 180 in both CD4+ and CD8+ T cell compartments and **c** comparative analysis of SARS-CoV-2 specific CD4+ and CD8+ T cell responses at day 28 (top panel) and day 180 (bottom panel) in both asymptomatic and mild groups (analysed as one group). Kruskal Wallis (one-way ANOVA on ranks) test, all P values are stated on plots. The boxplots all display the median values with the first and third quartile, and the whiskers represent the highest and lowest values no more than 1.5 times the interquartile range from the corresponding hinge.

We did not observe any differences in the magnitude of circulating FEC-specific (control) CD4+ or CD8+ T cells within the 6 months period (Supplementary Fig. 3C). Responses in both mild and asymptomatic groups were heterogeneous (Fig. 5a). In the asymptomatic group, at 28 days pso 7/8 (87.5%) made a CD4+ T cell response to at least one SARS-CoV-2 protein (excluding S1 and S2 where have previously reported finding responses in the majority of unexposed volunteers^5^) while 5/8 (62.5%) of them had CD8+ T cell response to at least one of M, NP, ORF3 or ORF8 proteins (Fig. 5a–c Supplementary Table 4). Most of this response was targeted to M and NP (Fig. 5a–c and Supplementary Table 4). At 180 days pso, 6/8 (75%) of recovered subjects had a CD4 + or CD8 + T cell response which was mostly focused on M, NP and ORF3. We observed no difference in the proliferative capacity of SARS-CoV-2-specific CD4 + and CD8 + T cells at 28- and 180-days post disease onset in the group with asymptomatic disease (*n* = 8) (Fig. 5a–c and Supplementary Tables 4 and 5, and Supplementary Data 1).

In the cohort with mild disease, at 28 days, T cell responses to at least one SARS-CoV-2 protein outside of spike region were observed in 42/49 (86%) for CD4+ T cells and 45/49 (91%) for CD8+ T cells. Similar to the asymptomatic cohort, these responses were focused on M, NP and ORF3 regions of SARS-CoV-2 (Fig. 5a–c, Supplementary Table 4). At 180 days after symptom onset, this frequency of people responding to at least one protein as above reduced to 37/49 (75%) within CD4+ T cells and 35/49 (71%) for CD8+ T cells with a focus on M, NP and ORF3 similar to CD4+ T cells (Fig. 5a–c and Supplementary Tables 4 and 5, and Supplementary Data 1). In the volunteers with mild disease, we found a significant reduction in the circulating frequencies of SARS-CoV-2-specific CD4+ and CD8+ T cells to all proteins except NP and ORF8 for CD4+ and ORF3 and ORF8 for CD8+ T cells by day 180 (Fig. 5a–c).

When we assessed the difference in the magnitude of the proliferative CD4+ and CD8+ T cell responses at 28- and 180 days pso in both asymptomatic and mild cases (analysed together as one group), we found significantly higher frequencies of SARS-CoV-2 specific CD4+ T cells compared to CD8+ responses at both timepoints in all proteins except NP and ORF8 for 28- and 180-days post symptom onset and ORF3 responses at 28 days post symptom onset only. Our data shows that the bias in antigen-specific responses to SARS-CoV-2 towards CD4+ T cells is maintained in the T cell memory compartment long after recovery from acute infection. Taken together, the results show that at 6 months post infection with SARS-CoV-2, convalescent subjects show diminished but detectable anti-SARS-CoV-2-specific memory T cells in both the CD4+ and CD8+ T cell compartments, with only 8/56 (14%) showing no proliferative response to any non-spike protein, suggesting durable immune response at least up to 6 months post initial infection.

### Integrative analysis to Identify immune and clinical parameters associated with disease severity

**Fig. 6:**
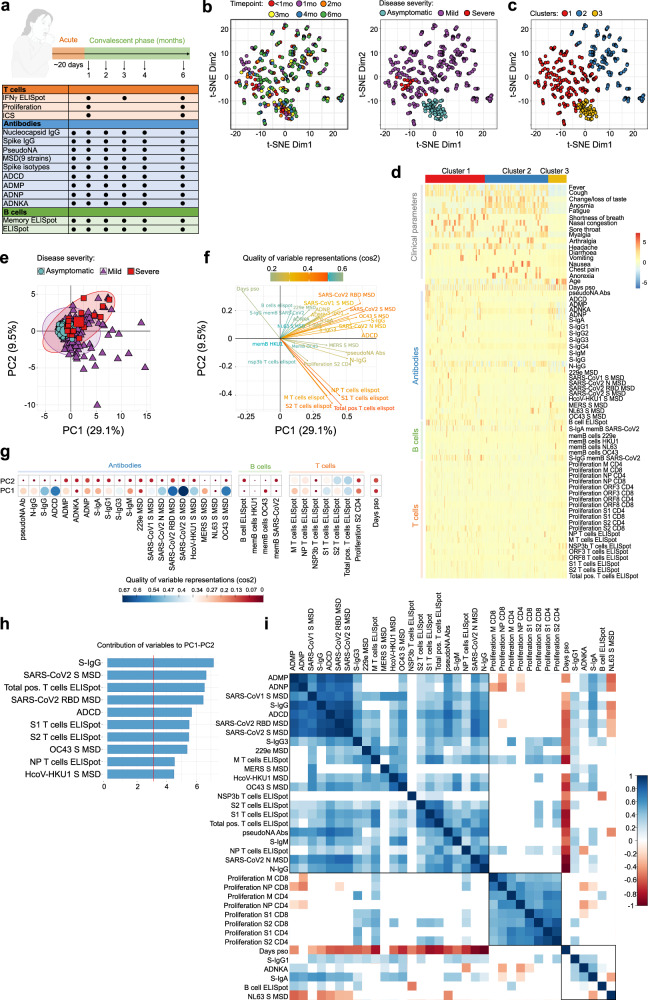
Integrative analysis of clinical and longitudinal immunological data reveals distinct immunophenotypic groups of SARS-CoV-2 infected individuals. **a** Clinical study overview. **b** t-SNE map of integrated clinical and immunological data colour-coded based on timepoint or disease severity. **c** Clustered t-SNE analysis. **d** Heatmap of clinical and immune parameters across three identified clusters. **e** PCA plot representing integrated immunological data, grouped based on the disease severity. Percentage indicates the variance explained by the principal component (PC). **f** Variable correlation plot. Positively correlated variables are grouped together, while negatively correlated variables are positioned on opposite quadrants. The distance between variables and the origin measures the quality of the variables on the factor map, while the colour indicated the quality of representations as cos2. **g** Quality of variable representations (colour-coded, cos2) and contributions of variables to principal components 1 and 2 (size of the circle). **h** Top 10 variables and their contribution to PC 1 and 2. **i** Correlations of immunological parameters with time component across samples. Spearman’s correlation coefficient (colour coded) and only significant values shown (adjusted for multiple testing using the Benjamini-Hochberg correction at the significance threshold FDR < 0.05). Black boxes indicate clusters (hierarchical clustering).

To further investigate the trajectory of cellular and humoral adaptive immune responses during SARS-CoV-2 infection and relationship with disease severity, we performed integrative analysis on aggregated immunological and clinical data from 433 samples obtained from 86 donors (12 asymptomatic, 66 mild, 8 severe) on 6 different timepoints (Fig. 6a). We investigated the trajectory of immune responses after SARS-CoV-2 infection and determined whether samples obtained from individuals with asymptomatic infection are more similar to samples obtained at later timepoints after infection in the individuals with mild, symptomatic disease. A t-distributed stochastic neighbour embedding (t-SNE) representation of integrated data revealed heterogeneity of immune responses in infected individuals, irrespective of days post symptom onset when these samples were collected (Fig. 6b, *left panel*). The majority of samples were separated between asymptomatic and mild individuals, while there was an overlap in similarity between individuals with mild and more severe disease (Fig. 6b*, right panel*). To further delineate differences in clinical and immunological parameters of SARS-CoV-2 infected individuals, we performed clustering analysis on the resulting t-SNE representations (Fig. 6c) and compared expression of 16 clinical and 49 immunological parameters to identify each of three clusters (Fig. 6d). This approach identified heterogeneity within the SARS-CoV-2 positive individuals with mild disease clustered in two groups (Fig. 6c, d, *clusters 1 and 2*). In cluster 1, the majority of samples displayed increased antibody and T cell responses in comparison to other clusters, and some individuals with mild infection that showed clinical and immunological similarity to severe COVID-19 patients (Fig. 6c, 6d, *cluster 1*). In contrast, cluster 2 contained individuals with lower overall antibody and T cell responses and all were from individuals with mild disease (Fig. 6c, 6d, *cluster 2*). Clinical parameters were driving a major separation between asymptomatic SARS-CoV-2 positive individuals from those with mild or severe disease (Fig. 6d, *cluster 3*).

To gain an insight into immunological differences between individuals with asymptomatic and mild infection, we performed principal component analysis (PCA) on the dataset containing only immunological parameters. The immunological parameters alone could explain 38.6% of variance between SARS-CoV-2 positive individuals, and separation of immune parameters was not driven by disease severity (Fig. 6e). Comparable to t-SNE analysis, samples from individuals with mild disease were separated into three major groups having either a distinct immunophenotype (immunophenotypic group 1) (Fig. 6e, *lower right quadrant*) or sharing immunological similarity with samples from individuals with severe (immunophenotypic group 2) (Fig. 6e, *upper right quadrant*) or asymptomatic disease (immunophenotypic group 3) (Fig. 6e, *center*). To reveal which parameters are driving the separation, we visualized the relationship between variables using a correlation analysis (Fig. 6f). T cell parameters were driving the separation of immunophenotypic group 1, while antibody responses separated immunophenotypic group 2 (Fig. 6f). The most important variables in explaining the variability between SARS-CoV-2 positive individuals in immunophenotypic group 1 were total IFN-γ ELISpot T cells, S1 and S2-stimulated IFN-γ ELISpot T cells, and anti-S IgG, anti-RBD IgG, ADCD, S-IgG from OC43 and HcoV-HKU1 in immunophenotypic group 2 that were correlated with principal components 1 and 2 (PC1-PC2) (Fig. 6g and h). The correlation plot revealed positive correlation between antibody responses, and negative correlation between T cell responses with the time when samples were obtained (Fig. 6f). To further examine these associations between immunological parameters, we performed correlation analysis, which confirmed strong positive correlation between antibody and T cells responses (Fig. 6i). The antibodies directed against N, S and RBD from SARS-CoV-2, were positively correlated with antibody functionality, such as pseudoneutralising capacity and ADCD, ADNP and ADMP, and positively correlated with IFN-γ ELISpot T cell responses against S1, S2 and N (Fig. 6i). The antibody responses to S protein from other circulating coronaviruses, such as SARS-CoV-1, MERS, HcoV-HKU1, 229e and OC43 were also contained in this cluster being positively correlated with antibody and T cell responses (Fig. 6i). This cluster was negatively correlated with time, confirming the observations from primary analysis (Fig. 6i). Notably, there was a negative correlation between NL63 S antibodies and S and RBD SARS-CoV-2 specific antibodies (Fig. 6i). There were other apparent relationships in two other clusters identified, that were not associated with time, including positive correlation between proliferating T cells stimulated with different SARS-CoV-2-specific peptides, and positive correlation between ADNKA and S-IgA and S-IgG1, while negative correlation with S-IgM (Fig. 6i).

The integrative analysis revealed three distinct immunophenotypic groups of SARS-CoV-2 infected individuals strongly connected to cellular and humoral immune profiling beyond the disease severity and clinical parameters.

### Identifying an early immunological signature associated with a durable immune response to SARS-CoV-2

**Fig. 7:**
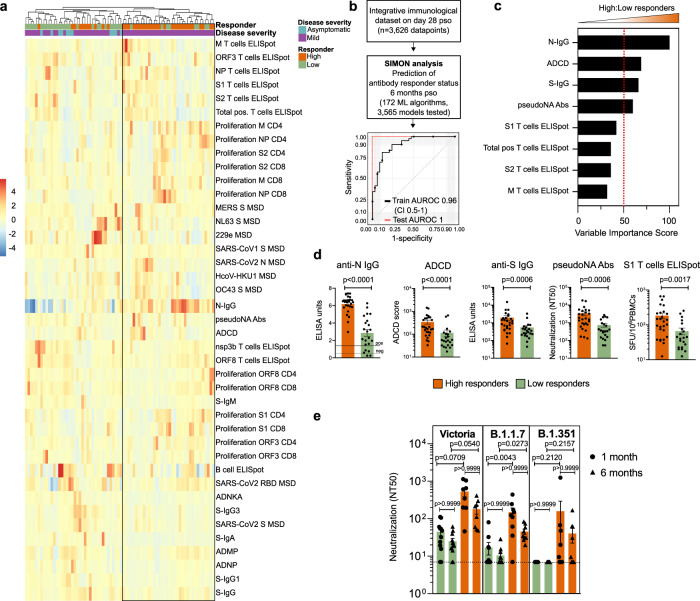
Early signature of durable SARS-CoV2 immune responses. **a** Hierarchical clustering heatmap of immune parameters on day 28 pso, grouping by responder status 6 months pso and disease severity. Results obtained using complete linkage agglomeration method, dendrogram ordered tightest cluster first. **b** Integrative immunological dataset containing 3,626 datapoints (49 features and 74 donors) was used for SIMON analysis to predict if the individual will generate high or low anti-N antibody responses 6 months pso. In total, 172 ML algorithms were tested and 3565 model built. ROC plot of the best performing model built with the svmPoly algorithm. Train AUROC (black line) is determined using 10-fold cross-validation and test AUROC evaluated on the independent test set (25% of the initial dataset). **c** Top variables that contribute to the model and are increased in high relative to low responders. **d** Frequency of selected variables on day 28pso (bars show mean with SEM). Mann–Whitney test (*p* < 0.05). (e) Neutralisation assay against wild-type SARS-CoV2 (Victoria), and two novel variants (B1.1.7 and B1.351) between high and low responders on two timepoints (one and 6 months pso). Plots show mean with SEM. A Kruskal-Wallis (one-way ANOVA on ranks), with Dunn’s multiple comparison test (*p* < 0.05) was performed (ns; adjusted *P* value > 0.9999).

To elucidate an early immunological signature that could predict whether an individual will mount a durable, high magnitude immune response against SARS-CoV-2 6 months after infection, we stratified SARS-CoV-2 infected individuals into high and low responders, based on the seropositivity status, using an anti-N IgG assay and seroconversion (anti-N IgG titre cut off of > = 1.4), which has recently been identified as a correlate of protection associated with reduced risk of SARS-CoV-2 re-infection 6 months after primary infection^32^. We then asked whether the components of cellular or humoral immunity within one month of infection (28 days pso) were predictive of the ability of individuals to develop serological response above this cut off that was sustained to 6 months pso. First, using an unsupervised machine learning approach, i.e., hierarchical clustering of integrated immunological data on day 28 pso, we identified two groups of SARS-CoV-2 infected individuals based on the serological response status 6 months pso (Fig. 7a). While the majority of SARS-CoV-2 infected individuals with mild disease generated an anti-N IgG titer cut off of > =1.4 antibody 6 months pso (high responders), there was a proportion of individuals with mild disease that failed to mount durable antibody response with anti-N IgG titres <1.4 6 months pso (low responders) (Fig. 7a). The majority of individuals with asymptomatic infection were low responders. High responders mounted stronger antibody responses, in particular N-IgG and pseudo-neutralising antibodies, and overall, stronger T cell responses, including IFN-γ-positive and proliferating T cells, than low responders at 28 days pso (Fig. 7a). Antibody responses to spike protein from 229e and NL63, B cell ELISpot and ADNKA were increased in low responders early after SARS-CoV-2 infection in comparison to high responders (Fig. 7a).

To further define the immunological features that can predict if the individual is on the trajectory to become a high or low responder, we used the SIMON supervised machine learning approach^30,31^. We generated 30 resamples and tested 3565 models using 172 machine learning algorithms (*Materials and methods*). The best performing model built using Sparse Partial Least Squares (sPLS) algorithm (train AUROC: 0.95 (CI 0.5-1) and test AUROC: 1) used only 8 out of 49 measured parameters on day 28 pso to predict if the individual will become high or low responder 6 months pso (Fig. 7b). The features that were contributing the most to this model included antibody responses to N and S, ADCD and pseudo-neutralising antibodies to SARS-CoV-2, and T cell IFN-γ ELISpot (S1/S2, M and total positive T cells) which were significantly increased in high responders 28 days pso compared to low responders (Fig. 7c, d). Together, these data indicate that early generation of antibodies with high binding, neutralising and effector function, and functional T cell responses following infection predicts the maintenance of immune responses 6 months after infection in an individual. Additionally, these findings suggest that a coordinated action of both T and B cells early after infection is required for establishment of durable antibody immunity.

The generation of durable and functional humoral and cellular immunity in SARS-CoV-2 infected individuals may provide protection against re-infection, including against variants of concern (VOCs). Thus, we assessed the neutralising antibody responses in high and low responders against the infecting (Victoria) strain and against variants B.1.1.7 and B.1.351 (Fig. 7e). High responders shown high neutralisation antibody titres against wild-type circulating SARS-CoV-2 (Victoria) strain, and against two novel variants, including B.1.1.7 (alpha) and B.1.351 (beta) (Fig. 7e). High responders also had significantly higher neutralising antibody titres against B.1.1.7 alpha variant one-month pso than low responders, and these higher neutralising antibodies were preserved 6 months pso, without significant decrease in the titers between both timepoints (Fig. 7e).

Altogether, these data suggest that generation of immunity to SARS-CoV-2 shows distinct trajectories following early priming, and early antibody responses are important to mediate durable immunity that may also neutralise novel variants.

## Discussion

Key questions on the trajectory of the SARS-CoV-2 specific immune response to past infection, and the maintenance of immune memory remain relevant even as highly effective vaccines are being rolled out worldwide. Firstly, there will always be a pool of unvaccinated people due to vaccine hesitancy or access difficulties, and this will include people who have had previous infection. Secondly, whilst 48% of the world’s population is estimated to have received at least one dose of vaccine by October 2021^33^, this is not evenly distributed across the globe so past infection may remain a cornerstone of population-level immunity in many regions. Thirdly, measuring immune responses to antigens not included in spike-containing vaccines is important for defining people with previous SARS-CoV-2 infection who are known to have enhanced vaccine responsiveness—evaluation of immune waning is essential to inform the use of these biomarkers^34,35^. Finally, understanding how the early immune response translates into durable immune responses against emerging variants of concern is crucial to accelerate predictions of population risk and to drive policy. In this manuscript, we characterise in detail the magnitude, function and maintenance of humoral and cellular T and B cell immune responses, the relationship between clinical and multi parametric immune data, the ability of antibodies to neutralise live SARS-CoV-2 virus 6 months after primary infection to variants of concern and provide insight into the early predictors of durable neutralising antibody responses after natural infection.

Compatible with other studies^9,12,36,37^, our data shows a peak of anti-NP and anti-S binding antibody (IgG) magnitude 28 days pso, with anti-NP responses declining over the next five months, although these responses remain above the threshold of detection in the majority. In contrast, anti-S IgG responses were well maintained, in keeping with the reported longer half-life for decay of anti-S IgG responses compared with anti-NP IgG responses^12^. B cell memory responses were also well maintained. Neutralisation measured by a pseudo-neutralisation assay showed a decline over time but was generally maintained six months following infection. High levels of neutralisation were seen earlier pso (from 7 days) than the observed rise in IgG levels (measured in binding assays), which may represent contributions to neutralising capacity from IgM^38^ and IgA^39^.

We assessed Fc-mediated antibody functionality including antibody dependent NK activation, phagocytosis and complement deposition and quantified IgG subsets over time. Previous studies have shown that early distinct antigenic targets and qualitative features of SARS-CoV-2-specific antibodies are associated with the severity of clinical disease^40,41^, whilst multifunctional antibody responses, and particularly ADCD and ADNP, following adoptive transfer of IgG from convalescent rhesus macaques have been shown to contribute to protection from SARS-CoV-2 challenge^42^. Furthermore, vaccine-induced Fc-mediated polyfunctionality has been observed following administration of efficacious vaccines in both macaque and human studies^43,44^. While the capacity of Fc receptor binding appears to be lower in convalescent individuals against VOCs, evidence is emerging of maintenance of vaccine-induced Fc-functional antibody properties against VOCs supporting resilience of humoral immunity against VOCs independent of neutralisation^45^. We show that Fc-mediated antibody functionality was maintained over the 6 months duration and was significantly associated with severe disease during primary infection. Assessing B cell polyfunctionality, we found this to be lower in those with asymptomatic infection, compared to those with mild disease early after infection (day 28), though by 6 months the profiles between the cohorts looked similar. The most notable changes were a reduction in IgM spike responses but a relative maintenance of IgG3 spike responses in the mild cohort that was not seen in the asymptomatic cohort. Currently, the contribution of these immune factors in providing protection from subsequent infection is not clear.

We showed significant heterogeneity between individuals in the magnitude of SARS-CoV-2 specific effector T cell responses over six months in an IFN-γ ELISpot assay, as previously reported^12,46,47^. The majority of people showed robust T cell responses in the first 28 days after infection, and there was a marked decline in T cell responses over 6 months. We used a flow cytometry based 7-day proliferation assay to show a dominant CD4+ T cell memory subset response. Although memory proliferative responses have been shown to ‘mature’ over time, particularly following vaccination^48,49^, we show that proliferative responses (both CD4+ and CD8+), targeting Spike, M, and NP decline markedly between day 28 and day 180. ICS analysis showed that CD4+ T cells were the dominant subset targeting S1, S2 and M antigens, whilst NP were targeted by both CD4+ and CD8+ T cells, and NSP3B was targeted by CD8+ T cells. Polyfunctional T cells, producing multiple cytokines, were generated at day 28 and retained out to 6 months with a partial decline.

We found that symptomatic infection is associated with more robust cellular and humoral immune responses compared to the asymptomatic group early after PCR + confirmed infection, and that people with asymptomatic infection had only low magnitude humoral and cellular responses six months after primary infection. An association between asymptomatic infection and lower antibody responses has been previously reported^50^, and we and others have shown a correlation between disease severity and higher levels of antibody and T cell responses in early disease^4,51^. Similar results have been reported in other disease settings including robust immune responses associated with disease severity in H1N1/09 influenza A^52^. In contrast, a previous prospective SARS-CoV-2 screening study has observed that asymptomatic infection is associated with highly functional cellular immune responses^53^. Although our study did not directly evaluate re-infection with SARS-CoV-2, our findings raise the possibility that people with asymptomatic SARS-CoV-2 infection may have less protection from re-infection after primary infection. The timing of infection onset in asymptomatic HCW (even though PCR+) cannot be precisely defined. As such, it is theoretically possible that the asymptomatic individuals in our study are later in their disease course at detection, though our integrative analysis did not support this possibility.

Our integrative analysis used over 70 immune and clinical parameters measured at multiple time points in 433 samples, leading to several interesting observations: Firstly, the t-SNE representation of integrated data revealed minimal clustering by time point, suggesting that heterogeneity of the immune response during SARS-CoV-2 infection arises independently of time after infection. The major separation of individuals with asymptomatic disease was driven by clinical parameters as expected. However, the mild cohort clustered into 2 immunophenotypic groups (not driven by clinical parameters), one of which shared a phenotype with the severe disease cohort. For the PCA, we excluded clinical parameters to focus the analysis on the assessment of immune responses and found that the total cohort separated into three immunophenotypic groups. The first immunophenotypic group was enriched with individuals with severe and mild disease and showed robust binding (anti-N and anti-S), functional (pseudoneutralising and ADCD/ADMP) antibody responses and memory B cell involvement. Immunophenotypic group 2 was composed of functional IFN-γ T cell responses and represented a unique subset of individuals with mild disease, early in the course of convalescence. The third immunophenotypic group showed lower overall antibody and T cell responses and contained individuals with mild disease and also the asymptomatic cohort, suggesting that some individuals may fail to develop robust antibody and T cell responses despite having symptomatic (mild) infection. We found that the immune phenotypes clustered independently from clinical disease severity and time after initial infection – this suggests that the immune response type is influenced by factors that are not directly associated with disease severity. It is currently unclear which factors drive these differential immune phenotypes, but the possibilities include clinical demographic factors, viral factors during primary infection including infecting dose, or immune factors that may be driven by genetic differences or prior exposures to seasonal human coronaviruses.

Our second key finding is the identification of an early immunological signature that defines the trajectory of the SARS-CoV-2 immunity following past infection based on antibody titers associated with reduced risk of SARS-CoV-2 re-infection 6 months after the primary infection^34^. High anti-N IgG, along with more robust overall T cell responses at baseline with a low response to seasonal coronaviruses (NL63 and 229e) dominated in the high responder group, whilst low responders had lower anti-N IgG and overall T cell responses and had more pronounced cross-reactive seasonal CoV responses (NL63 and 229e) at baseline. The final major finding was the ability to predict whether an individual will generate SARS-CoV-2-specific antibody immunity 6 months post infection based on the early immunological signature one month after infection. The predictive model built by SIMON supports a link between both arms of the immune response—cellular and humoral immunity—in early responses to natural infection with the durability of the SARS-CoV-2 antibody immunity. This early immunological signature may determine essential differences of the trajectory that each individual will take after SARS-CoV-2 infection, including also neutralisation of VOC. We did not examine responses against the B.1.671.1/delta VOC, which had not yet emerged at the time of this study. Further studies by our group and others indicate that following natural infection with the early pandemic strain (Victoria) or alpha variant, neutralising antibodies against B.167.2 are reduced but do not show the widespread escape seen for B.1.351^29^, and T cell responses are well maintained to VOC after past infection and/or vaccination^28,54,55^.

A limitation to the interpretation of data in this study is that we were not able to determine the contribution of immune parameters to subsequent protection from SARS-CoV-2 infection or from subsequent infection and severe disease since this cohort of HCW received COVID-19 vaccines shortly after the 6 month follow up. However, published data suggests that titres of anti-N Abs do predict risk from subsequent infection^9^, and further inferences may be made from past studies of seasonal coronaviruses that have reported frequent re-infections in association with waning antibody responses^17–19^ suggesting that protective immunity against infection in all coronaviruses is short lived. Nevertheless, in a series of human challenge studies^56^, Callow and colleagues showed that experimental reinfection with seasonal coronavirus 229E induced memory responses associated with a reduction of both viral shedding and symptoms - suggesting prior infection attenuates the course of subsequent infections. Sera from SARS-CoV-2 uninfected individuals has been shown to exhibit neutralizing activity against SARS-CoV-2 indicating that previous exposures to seasonal coronaviruses may protect against SARS-CoV-2 infection^57^.

Importantly, immune parameters that protect from re-infection may differ from those that protect from severe disease after subsequent infection. Recent studies have demonstrated that the presence of pre-existing cross-reactive CD4+ and CD8+ T cells is associated with reduction in the COVID-19 severity, supporting a protective role for T cells against disease^58,59^. This is further exemplified in emerging data seen following SARS-CoV-2 vaccination, where memory T and B cell responses are preserved and vaccination protects from severe disease in spite of waning anti-spike Ab responses^60^. In our cohort, although immune responses generally declined over time, T and B cell polyfunctional responses remained detectable in the majority of people, and these may serve to protect from either re-infection or severe disease. Moreover, correlation analyses revealed positive associations between spike and nucleocapsid T cell and antibody responses and cross-reactivity to other coronaviruses, substantiating the findings that SARS-CoV-2 immunity associated with reduction in the COVID-19 severity may be defined by immunocompetence and previous exposures to circulating seasonal coronaviruses.

Overall, our data reveal the highly variable range of immune responses after SARS-CoV-2 infection and suggest that immune events primed during early SARS-CoV-2 infection may define the subsequent trajectories leading to the effective maintenance or loss of long-term SARS-CoV-2 immune responses including neutralising antibodies. We show that people with asymptomatic infection had lower responses at all time points across many of the immune parameters measured. Our data suggests that previous infection may not give ongoing protection against VOC, as neutralising Abs to these wane when assessed 6 months later. Maintenance of immune memory over time is critically required for the effective neutralisation of VOC that is most likely to confer sterilising immunity, whilst other immune mechanisms including non-neutralising antibodies and T cells may contribute to protection against severe disease, including for VOC^61–64^. Our data supports the ongoing COVID19 vaccine efforts to maximise the generation of a multi-functional immune response to SARS-CoV-2, irrespective of prior infection status.

## Methods

### HCW volunteer recruitment and ethics

We sampled seventy-eight HCW at five or six time points each, over six months. HCWs were recruited from Oxford University Hospitals NHS Foundation Trust after a positive SARS-CoV-2 PCR test^2^ in April-May 2020, including 66 volunteers with symptomatic disease (fever, shortness of breath, cough, loss of taste or smell, sore throat, coryza or diarrhoea) and 12 asymptomatic HCW who did not report any symptoms of COVID-19 in 2020 prior to staff screening or in the seven days following testing positive. The age, sex and ethnicity of the HCW are shown in Supplementary Table 1. Blood samples were acquired at multiple timepoints over 6 months (acute[range:1–20; median 13], 28 days [21–41; median 29], 56 days [42–73; median 56], 90 days [74–104; median 90.5], 120 days [110–140; median 120], and 180[160–200; median 178]) from onset of symptoms in the symptomatic group and from the date of positive PCR test for asymptomatic people diagnosed on screening. Nine hospitalised patients with severe disease were included for comparative analysis. All subjects were seropositive for anti-spike IgG antibodies by ELISA. Mild and asymptomatic participants were recruited under ethics approved by the research ethics committee (REC) at Yorkshire & The Humber–Sheffield (GI Biobank Study 16/YH/0247). Participants with severe disease were recruited after consenting into either the CMORE study protocol (research ethics committee (REC): Northwest–Preston, REC reference 20/NW/0235) and/or Sepsis Immunomics protocol [Oxford Research Ethics Committee C, reference 19/SC/0296]. The study was conducted according to the principles of the Declaration of Helsinki (2008) and the International Conference on Harmonization (ICH) Good Clinical Practice (GCP) guidelines. Written informed consent was obtained for all participants enroled in the study.

### Isolation of peripheral blood mononuclear cells (PBMC), plasma and serum

PBMCs and plasma were isolated by density gradient centrifugation from blood collected in EDTA tubes, and serum was collected in a serum-separating tube (SST, Becton Dickinson) as previously described^5^. Briefly, PBMCs were isolated by density gradient centrifugation using Lymphoprep^TM^ (*p* = 1.077 g/ml, Stem Cell Technologies), washed twice with RPMI 1640 (Sigma, St. Louis, MO, USA) containing 10% heat-inactivated FCS (Sigma), 1 mM Pen/Strep (100 U/mL) and 2 mM L-glutamine (100 ug/mL) (Sigma) or AutoMACS Rinse Buffer and resuspended in R10 or AutoMACs Rinse Buffer and counted using the Guava® ViaCount^TM^ assay on the Muse Cell Analyzer (Luminex Cooperation). PBMCs were frozen and stored in liquid nitrogen. To obtain plasma, the uppermost fraction following the initial Lymphoprep centrifugation above was collected and centrifuged at 2000*g* for 10 min to remove platelets before storage at −80 °C. Donor blood was collected in a serum-separating tube (SST, Becton Dickinson) which was centrifuged at 2000*g* for 10 minutes. Serum was removed and stored at −80 °C.

### T cell assays

T cell assays including interferon-gamma (IFN-γ) Enzyme-Linked immunospot (ELISpot) assay, 7-day proliferation assay and intracellular staining were performed as described^5^. For all T cell assays we used SARS-CoV-2 spanning Spike (S1 and S2), membrane (M), nucleocapsid protein (NP), the X-domain of non-structural protein 3 (NSP3B), open reading frames 3 and 8 (ORF3 and ORF8) overlapping peptide pools (OLPs) (Supplementary Table 6). OLPs were either 15nmers overlapping by 10 or 18nmers overlapping by 11. For positive control we used cytomegalovirus, Epstein-Barr virus, influenza and tetanus antigens (CEFT) peptide pool (2 µg/ml, GenScript, Piscataway, NJ, USA).

#### IFN-γ Enzyme-Linked immunospot (ELISpot) assay

96-well Multiscreen-I plates (Millipore, UK) were coated for 3 hours with 10 μg/ml clone 1-D1K (Mabtech, AB, Sweden) at room temperature. PBMC were added in duplicate wells at 2 × 10^5^ cells in 50 μl per well and stimulated with 50 μl of SARS-CoV-2 peptide pools (2 ug/ml per peptide). R10 with DMSO (final concentration 0.4%, Sigma) was used as negative control. After 16-18 hours at 37 °C PBMC were removed and secreted IFN-γ detected using anti-IFN-γ biotinylated mAb at 1 μg/ml (7-B6-1-biotin, Mabtech) for 2–3 h, followed by streptavidin alkaline phosphatase at 1 μg/ml for 1-2 h (SP-3020, Vector Labs). The plates were developed using BCIP/NBT substrate (Pierce) according to the manufacturer’s instructions. ELISpot plates were read using an AID ELISpot Reader (v.4.0). Results were reported as spot-forming units (SFU)/10^6^ PBMC. Background (mean SFU in negative control wells) was subtracted from antigen stimulated wells to give the final result. Only assays where the background was 50 SFU/10^6^ or below were accepted as valid. The cut-off threshold for a positive result was the mean of the negative control well plus 2 times the standard deviation. The lower limit of quantification (LLOQ) for this assay is 2.5 SFU/10^6^ PBMC, values below this were assigned a value of 1.

#### T cell proliferation assay

T cell proliferation assay was performed using fresh (*n* = 3) or cryopreserved PBMC (*n* = 111) and CellTrace® Violet (CTV, Life Technologies) labelling (final concentration of 2.5 μM). Labelling with CTV was done in PBS for 10 min at room temperature following which the reaction was stopped using ice-cold fetal bovine serum (FBS). The CTV-labelled PBMC were then plated at 0.25 × 10^6^ cells per well of a 96 well round bottom plate in RPMI supplemented with 10% human blood group type AB serum (Sigma), 1% 1 mM Pen/Strep and 1% 2mM L-glutamine and stimulated with peptide pools from SARS-CoV-2 spanning Spike (S1 and S2), M, NP, ORF3 and ORF8, and FEC-T (1 μg/ml per peptide). For controls, media containing 0.2% DMSO (Sigma) representing DMSO content in peptide pools was used as a negative control and phytohemagglutinin L (PHA-L, Sigma) at a final concentration of 2 ug/ml was used as positive control. Cells were then incubated at 37 °C, 5% CO2, 95% humidity for 7 days with hemi-depletion of media on day 4. On day 7, cells were stained for analysis on the flow cytometer. Briefly, PBMC were resuspended in cell staining buffer (Biolegend, San Diego, CA, USA) and incubated for 20 min with live/dead near-infrared (Invitrogen, Carlsbad, CA, USA). This was washed off and the cells were incubated with fluorochrome-conjugated primary human-specific antibodies for CD3 (1:100 dilution), CD4 (1:200 dilution) and CD8 (1:200 dilution) in cell staining buffer (Biolegend, San Diego, CA, USA) for 30 min at 4 °C. This was followed by a wash with cell staining buffer and then fixation with 4% paraformaldehyde (PFA, Sigma). Cells were stored at 4 °C in the dark until data acquisition on a MACSQuant 10. Responses above 1% were considered true positive. All data is reported as background subtracted data for each volunteer.

#### Intracellular cytokine staining

PBMC were thawed and rested overnight in R10 media (1 million cells for peptide stimulation and 500,000 for DMSO and PMA controls) in round bottom plates. Afterwards, cells were stimulated with SARS-CoV-2 peptide pools (2 ug/ml) (Supplementary Table 6), R10 media containing DMSO (0.1%, Sigma) for negative controls and PMA (0.05 ug/mL) with ionomycin (0.5 ug/mL, Sigma) as a positive control. CD107a BV421 1:100 dilution (clone H4A3, BD Biosciences), monensin (Biolegend) and Brefeldin A (MP Biomedicals) were added to cultures at a final concentration of 0.04 ug/mL, 0.16 uM, and 10 ug/mL respectively, and cells were incubated for 6 h at 37 °C, 5% CO_2_, 95% humidity. PBMC were then washed with PBS and stained with LIVE/Dead Fixable Aqua stain (Life Technologies) at a 1:400 dilution in PBS and stained for 20 min at room temperature. Cells were washed with PBS and resuspended in Cytofix/Cytoperm (BD Biosciences) and incubated for 20 min at 4 °C. Afterwards, cells were washed twice with BD Perm/Wash buffer (BD Biosciences). Cells were then stained with the following panel of antibodies in Perm/Wash buffer for 20 min at room temperature: CD3 APC Fire 750, 1:00 dilution (clone SK7, Biolegend), CD4 PE Dazzle 594, 1:200 dilution (clone RPA T4, Biolegend), CD8 PerCpCy5.5, 1:200 dilution (clone RPA T8, Biolegend), CD154 PE-Cy7, 1:50 dilution (clone 24-31, Biolegend), IFN-γ APC, 1:50 dilution (clone B27, BD Biosciences), IL-2 PE, 1:100 dilution (clone MQ1-17HI2, Biolegend), TNF-α FITC 1:100 dilution (clone Mab11, BD Biosciences). Cells were then washed twice with Perm/Wash buffer before resuspending the cells in PBS and running them on a BD LSR II. OneComp compensation beads were used (Life Technologies) as were rainbow fluorescent particles (mid-range intensity) (Biolegend) to calibrate the LSR II before acquisition.

### Antibody and B cell assays

#### Anti-spike and anti-nucleocapsid total IgG (ELISA/EIA)

Standardised total anti-spike IgG ELISA^65^ and anti-spike subclass and isotype ELISAs^44,66^ were performed. In brief, ELISA plates were coated with 2 µg/mL of full-length trimerised SARS-CoV-2 spike glycoprotein protein overnight at 4 °C and blocked with casein in PBS. Plasma samples were diluted in PBS and tested in triplicate. Goat anti-human IgG conjugated to alkaline phosphatase was added as the secondary antibody, and plates were developed using 4-nitrophenyl phosphate in diethanolamine substrate buffer. Plates were read at 405 nm, and standardised ELISA units (EU) were determined using a 4-parameter logistic model and various pre-determined control cut-offs (Gen5 v3.09, BioTek). Plate washing in-between each step was undertaken using 0.05% Tween-20 in PBS.Serology for IgG to SARS-CoV-2 nucleocapsid protein was performed using the Abbott Architect i2000 chemiluminescent microparticle immunoassay (Abbott, Maidenhead, UK) and carried out according to manufacturer’s instructions using serum. The manufacturer threshold for confirming detection of antibodies is ≥1.40 arbitrary units. Levels between 0.50-1.39 arbitrary units designate equivocal levels (Abbott Diagnostics Product Information Letter PI1060-2020). Values below 0.5 were set to half the LLOQ (i.e. 0.25).

#### Anti-spike subclass and isotype ELISAs

Both isotype and subclass standardised and OD ELISAs were performed as described previously^44^. In brief, ELISA plates were coated with 5 µg/mL of full-length trimerised SARS-CoV-2 spike protein for overnight incubation at 4 °C. Following washing with 0.05% Tween-20 in PBS (PBS/T) plates were blocked with casein in PBS for non-specific binding. In the next step plasma samples were diluted in casein in PBS, as well as positive, negative controls and ten-point standard curve. Plates were incubated for 2 h at 37 °C with 300 rpm shaking and following washing with PBS/T samples were further incubated with mouse anti-human IgG1 hinge-AP, mouse anti-human IgG3 hinge-AP, goat anti-human IgA-AP and goat anti-human IgM-AP (Southern Biotech) for 1 h at 37 °C with 300 rpm used for detection and the optical density at 405 nm was measured until the internal control reached an OD_405_ of 1. For detection of anti-spike IgG2 and IgG4 steps modified as follows: (1) Plates were additionally coated with commercially available human immunoglobulin control (recombinant human IgG2 lambda or recombinant human IgG4 lambda (Bio-Rad)) to serve as internal controls, (2) Mouse anti-human IgG2 Fd-AP or mouse anti-human IgG4 Fc-AP (Southern Biotech) were used, and (3) Optical density at 405 nm was measured using an ELx808 absorbance reader (BioTek) until the immunoglobulin control reached a specified OD405. Standardised ELISA units (EU) were determined using a 4-parameter logistic model and various pre-determined control cut-offs (Gen5 v3.09, BioTek) while for OD ELISAs negative cut offs were calculated using the formula: mean + 7.858 × standard deviation of the OD405 readings of the pre-pandemic negative-control serum samples, where 7.858 is the standard deviation multiplier with a 99.9% confidence level for *n*  =  5 controls as detailed in^44,66^. LLOQ were 11, 12, 12, 18, 0.2, 8 and 0.2 EU for total IgG, IgM, IgA, IgG1, IgG2, IgG3 and IgG4, respectively—values below LLOQ were set to half the LLOQ.

#### MSD Common Cold Coronaviruses

A multiplexed MSD immunoassay (MSD, Rockville, MD) was used to measure the IgG responses to SARS-CoV-2, severe acute respiratory syndrome coronavirus-1 (SARS-CoV-1), MERS-CoV and seasonal CoVs (human coronavirus (HCoV)-OC43, HcoV-HKU1, HcoV-229E, HcoV-NL63). A MULTI-SPOT® 96-well, 10 Spot Plate was coated with three SARS CoV-2 antigens (S, RBD, N), SARS and MERS-CoV spike trimers, as well as spike proteins from seasonal CoV HCoV-OC43, HCoV-HKU1, HCoV-229E, HCoV-NL63 and bovine serum albumin. Antigens were spotted at 200 − 400 μg/ml (MSD® Coronavirus Plate 3). Multiplex MSD Assays were performed as per the instructions of the manufacturer. To measure IgG antibodies, 96-well plates were blocked with MSD Blocker A for 30 min. Following washing with washing buffer, our samples diluted 1:500-1:5000 in diluent buffer, as well as the reference MSD standard and internal MSD controls were added to the wells. After 2-hour incubation and a washing step, detection antibody (MSD SULFO-TAG™ Anti-Human IgG Antibody, 1/200) was added. Following washing, MSD GOLD™ Read Buffer B was added and plates were read using a MESO® SECTOR S 600 Reader. The standard curve was established by fitting the signals from the standard using a 4-parameter logistic model. Concentrations of samples were determined from the electrochemiluminescence signals by back-fitting to the standard curve. They were multiplied by the dilution factor and expressed in Arbitrary Units/ml. LLOQ were 1160.3, 1169.0 and 3873.5 AU/ml for SARS-CoV-2 spike, SARS-CoV-2 RBD and SARS-CoV-2 NP, respectively—values below the LLOQ were set to half the LLOQ.

#### Microneutralisation Assay (MNA)

Microneutralisation Assay (MNA) was performed to determine the concentration of antibody that produces a 50% reduction in infectious focus-forming units of authentic SARS-CoV-2 viral isolates in Vero CCL81 cells. Prototype isolate (PANGO lineage B) was Victoria/01/2020^67^ received at Passage(P)3 from Public Health England (PHE) Porton Down (after being supplied by the Doherty Centre Melbourne) in April 2020, passaged in VeroE6/TMPRSS2 cells, used here at P5, and confirmed identical to GenBank MT007544.1, B hCoV-19_Australia_VIC01_2020_ EPI_ ISL_ 406844_ 2020-01-25. B.1.1.7 (20I/501Y.V1.HMPP1) isolate, H204820430, 2/UK/VUI/1/2020, received in Oxford at P1 from PHE Porton Down in December 2020, passaged in VeroE6/TMPRSS2 cells (NIBSC reference 100978), used here at P4. B.1.351 (20I/501.V2.HV001) isolate was received at P3 from the Centre for the AIDS Programme of Research in South Africa (CAPRISA), Durban, in Oxford in January 2021, passaged in VeroE6/TMPRSS2 cells (NIBSC reference 100978), used here at P4. For all isolates, identity was confirmed by deep sequencing at the Wellcome Trust Centre for Human Genetics, University of Oxford. Quadruplicate serial dilutions of serum were preincubated with 100-200 FFU (20 μL) of SARS-CoV-2 for 30 minutes at room temperature. After pre-incubation, 100 μL of Vero CCL81 cells (4.5 × 10^4^) were added and incubated at 37 °C, 5% CO_2_. After 2 h, 100 μL of a 1.5% carboxymethyl cellulose-containing overlay was applied to prevent satellite focus formation. Eighteen (B.1.351) or 23 h (B, B.1.1.7) post-infection, the monolayers were fixed with 4% paraformaldehyde, permeabilized with 2% Triton X-100 and stained for the nucleocapsid antigen or spike (S) antigen using monoclonal antibodies (mAbs) EY 2 A and EY 6 A, respectively^68^. After development with a peroxidase-conjugated antibody and TrueBlue peroxidase substrate, infectious foci were enumerated by ELISpot reader. Data were analysed using four-parameter logistic regression (Hill equation) in GraphPad Prism 8.3.

#### Monogram Bioscience pseudotype neutralisation assay (PseudoNA)

PseudoNA was performed using a lentivirus-based SARS-CoV-2 pseudovirus particle expressing spike protein^65^. Briefly, plasma samples were heat inactivated at 56 °C for one hour and diluted in a 9 serial three-fold dilution series starting at 1:40 in cell culture medium. Each sample dilution was mixed with 10^5^ relative light units of a lentivirus-based SARS-CoV-2 pseudovirus particle virus. As an internal assay control, an irrelevant pseudotyped virus was also incubated with test samples. The pseudotyped virus and sample mixtures were incubated for one hour at 37 °C and HEK 293 ACE2-transfected cells were added to each well, which were then incubated for a further 60–80 h at 37 °C. Luciferase expression was determined, and neutralisation titres are reported as the reciprocal of the plasma dilution conferring 50% inhibition (ID50) of pseudovirus infection. %Inhibition = 100%–(((RLU(Vector + Sample+Diluent)–RLU(Background))/(RLU(Vector + Diluent)–RLU(Background))) x 100%). Inter-assay variation was controlled for by monitoring results acquired from one positive control, one negative control and six patient specimens. The LLOQ for this assay is a titre of 1:40, values below this were set to half LLOQ (i.e. 20).

#### Spike-specific SARS-CoV-2, OC43, HKU1, 229E and NL63 IgG^±^ and IgA^±^ B cell memory ELISPOT

PBMCs were adjusted to 2 × 10^6^ cells/ml in complete media and 2 × 10^5^ cells/per well were added to a 96-well round bottomed plate with an equal volume of complete media supplemented with 1 µg/ml R848 and 10 ng/ml of recombinant IL-2, each from the Mabtech Memory B-cell Stimpack. The cells were cultured for 3–3.5 days at 37 °C in 5% CO_2_. Following polyclonal stimulation, the cells were harvested, washed twice in complete media and counted. Mabtech flurospot plates were activated with 35% ethanol and coated with the relevant spike glycoprotein (SARS-CoV-2 at 10 µg/ml, OC43 at 10 µg/ml, NL63 at 15 µg/ml, HKU1 at 5 µg/ml and 229E at 10 µg/ml, all diluted in PBS). Control wells were coated with tetanus toxoid (5 µg/ml), capture mAbs anti-human IgG (Mabtech MT91/145) and PBS as a negative control. Following incubation for 16-20 h at 4 °C, the plates were washed five times with PBS and blocked for ≥30 min with complete media. The harvested PBMCs were adjusted so that 2 × 10^5^ cells were added to the spike- and tetanus toxoid-coated, and PBS wells while 2 × 10^4^ cells were added to the IgG positive control wells. All cells were incubated for ≥16 h at 37 °C, plates were washed five times with PBS and detection mAbs IgG-550 (Mabtech MT78/145) and IgA (Mabtech MT20-490) were diluted 1:500 in 0.5% BSA in PBS and added to plates for two hours at room temperature. Following five washes in PBS, fluorescent enhancer solution was added to each well for 15 min at RT in the dark. Plates were decanted and blotted dry and stored in the dark. Spot forming units were enumerated using AID ELISpot 8.0 software on the AID ELR08IFL reader. The LLOQ for these assays is 1 SFU, values below this were set to half LLOQ (i.e. 0.5).

### Antibody-dependent effector functions

For antibody-dependent effector functions, the spike-specific antibody-dependent effector functions, natural killer cell activity (ADNKA), neutrophil phagocytosis (ADNP) and monocyte phagocytosis (ADMP) were performed as previously described^44^. To prepare beads for ADNP and ADMP, red fluorescent (580/605) NeutrAvidin-labelled microspheres (Thermo Fisher, F8775) were freshly coupled to biotinylated SARS-CoV-2 spike protein for each assay. Spike protein (at a concentration of 0.388 µl/ml) was added to the beads at a 3:1 ratio and incubated for 2 h at 37 °C. Beads were washed twice with 0.1% BSA and diluted 100-fold in 0.1% BSA. 10 µl was added to each well in the ADNP and ADMP assays. For all three assays, normalized phagocytic scores were calculated by multiplying the percentage of bead-positive cells with the MFI of the events within the bead-positive cell gate and normalizing against a QC sample. As multiple plates were run during an experiment, plates failed if any of the QC sample averages were greater than two standard deviations above the mean of that particular QC across plates. In addition, samples were excluded from further analysis if the replicates showed a coefficient of variation of over 25%. All data were derived from one experiment. The LLOQ for ADNP assay is 0.033, ADMP 0.23, and ADNKA 3.5, values below these were set to half the LLOQ.

#### ADNP assay

Whole donor blood, collected in sodium heparin tubes, was treated with ammonium–chloride–potassium lysing buffer (Thermo Fisher, A1049201) for 5 minutes followed by centrifugation to collect white blood cells. Cells were washed with DPBS (Sigma, D8537), counted and adjusted to 2.5 × 105 cells per ml in medium consisting of RPMI 1640 medium (Sigma, R5886) supplemented with 100 U ml−1 penicillin–streptomycin (Sigma, P4458) and 20 mmol/L l-glutamine (Sigma, G7513). Serum diluted 100-fold in RPMI was added to antigen-coupled beads in a 96-well plate and incubated for 2 h at 37 °C. All samples were assayed in duplicate, and each plate contained two QC samples in addition to appropriate negative controls. Wells were washed with DPBS, and 50,000 white blood cells were added to each well followed by a further one hour incubation at 37 °C. Cells were then stained using a cocktail of mouse anti-human CD3 Alexa Fluor 700 (BD Pharmingen, clone UCHT1, nos. 557943 and 9185576; 1:80 dilution), mouse anti-human CD14 APC Cy7 (BD Pharmingen, clone MΦP9, nos. 557831 and 0044497; 1:80 dilution) and mouse anti-human CD66b Pacific Blue (BioLegend; clone G10F5, nos. 305112 and B285068; 1:80 dilution) and incubated for 15 minutes at room temperature in the dark. Following washing and fixation using 4% paraformaldehyde (Santa Cruz Biotechnology, SC-281692), cells were analysed by flow cytometry (BD, Fortessa X20). Data were analysed with FlowJo (BD; version 10), using a gating strategy to select neutrophils. Neutrophils were gated based on forward and side scatter then doublets excluded. Furthermore, T cells and monocytes were excluded using a double-negative gate for CD3 and CD14. The final neutrophil gate was based on CD66b positivity, after which bead-positive cells were gated. In all cases, there was a clear separation between positive and negative populations.

#### ADMP assay

Human monocytic THP-1 cells (American Type Culture Collection) were grown and maintained using supplier instructions. Serum was diluted 1:4,000 in RPMI, added to antigen-coupled beads in a 96-well plate, and incubated for two hours at 37 °C. All samples were assayed in duplicate, and each plate contained two QC samples in addition to appropriate negative controls. At the end of the two hours incubation period, wells were washed with RPMI and 25,000 THP-1 cells diluted in medium consisting of RPMI 1640 medium (Sigma, R5886) supplemented with 100 U/mL penicillin–streptomycin (Sigma, P4458) and 20 mmol/L l-glutamine (Sigma, G7513) were added to each well. Plates were then incubated for 18 h at 37 °C. Cells were then washed with PBS and fixed using 4% paraformaldehyde before analysis by flow cytometry (BD, Fortessa X20). Data were analysed with FlowJo (BD, version 10). THP-1 cells were gated based on forward and side scatter to exclude debris then doublets excluded and bead-positive cells gated. There was a clear separation between the positive and negative population.

#### ADNKA assay

SARS-CoV-2 spike protein in carbonate/bicarbonate solution (2.5 µg/ml) was added to 96-well Nunc MaxiSorp ELISA plates and incubated for 16 h at 4 °C. Plates were washed six times with PBS and blocked with 5% BSA in PBS for one hour at 37 °C. Plasma samples were added neat and in duplicate, and plates were incubated for two hours at 37 °C. Following another wash step, 10^5^ natural killer NK-92 cells expressing human CD16 (PTA-8836 cell line, American Type Culture Collection) described by^69^ were added to each well with brefeldin A (10 µg/mL; Sigma Aldrich), GolgiStop (BD Biosciences) and CD107a (1:20 dilution; PE, clone H4A3, BD Biosciences). Plated cells were incubated for five hours at 37 °C and then transferred to V-bottom plates, incubated with fixable LIVE/DEAD staining (1:500 dilution; R780, BD Biosciences) and fixed. Data was acquired using a BD Fortessa and percentages of CD107a expressing NK cells relative to control wells with spike protein and blocking buffer only were determined using FlowJo Software (version 10.7.1). To assess inter-assay variation, both a pre-pandemic pool of three donors and a pool of six hospitalised SARS-CoV-2-infected individuals were plated in triplicate on each plate.

#### Antibody-dependent complement deposition (ADCD) assay

SPHERO^TM^ Carboxyl magnetic blue fluorescent beads (Spherotech, USA) were coupled with SARS-CoV-2 whole spike protein (Lake Pharma, USA) using a two-step Sulpho-NHS/EDC process. Briefly, 5 million beads were washed with 82 mM sodium phosphate buffer pH 6.2, prior to activation in the same buffer containing 1.24 mg each of N-hydroxysulfosuccinimide and 1-ethyl-3-[3- dimethlyaminopropyl]carbodiimide-HCl). After 20 min activation, the beads were washed in coupling buffer of 50 mM 2-(N-morpholino) ethanesulfonic acid (MES) pH 5.0 and resuspended in MES buffer containing 14.5 µg antigen for 2 h on a rotational mixer. Finally, beads were washed three times with PBS containing 2% BSA and 0.05% sodium azide, pH7.4, and resuspended in the same buffer overnight. Beads were washed and resuspended in a storage buffer of PBS with 0.05% sodium azide, pH7.4, until use. Heat-inactivated test serum (3 µl, in duplicate) was added to 27 µl assay blocking buffer (PBS + 2% BSA:BB) and 10 µl taken for serial 3-fold dilutions to give final dilutions of 1:20, 1:60, 1:180, 1:540. 20 µl of spike-coated magnetic beads (50 beads per µl) was added, and the mixture incubated at 25 °C for 30 min with shaking at 900 rpm. The beads were washed twice in 200 µl wash buffer (BB + 0.05% Tween-20: WB) and then resuspended in 50 µl BB containing 10% IgG- and IgM-depleted human plasma, prepared as described previously^70^ and incubated at 37 °C for 15 min with shaking at 900 rpm. Beads were next washed twice with 200 µl WB and resuspended in 100 µl FITC-conjugated rabbit anti-human C3c polyclonal antibody (Abcam, UK) and incubated at room temperature in the dark. After two more washes with 200 µl WB, the samples were resuspended in 40 µl Hank’s Balanced Salt Solution and analysed on the IntelliCyt® iQue Screener PLUS platform (Sartorius, Germany) and ForeCyt®t 8.0 software. For each sample, a minimum of 100 beads were collected and complement activation units (CAU) calculated using a 12-point standard curve of the Anti-SARS-CoV-2 Antibody Diagnostic Calibrant (20/162 NIBSC, UK), with the calibrant standard assigned 1000 CAU. The LLOQ for this assay is 10, values below this were set to half the LLOQ.

### Integrative analysis using unsupervised and supervised machine learning in SIMON

The integrative analysis using unsupervised and supervised machine learning was performed using SIMON (Sequential Iterative Modeling “Over Night”) software^30,31^.

#### Generation of the integrated dataset and data pre-processing

The integrated dataset was generated using the standard extract-transform-load (ETL) procedure, as described^71^. Briefly, primary analysis datasets which included total of 29 csv files across 14 assays and clinical data were merged using donor-specific variable (Donor ID). The outcome of immune response durability was calculated based on the titre of the anti-N specific antibodies measured 6 months post symptoms onset (pso), and individuals with anti-N antibody titre ≥ 1.4 were labelled as high responders, while individuals having anti-N antibody titre below 1.4 were low responders, as this titer cut-of values were previously identified to be associated with the reduced risk of SARS-CoV-2 re-infection 6 months after the primary infection^34^. The responder status was expressed as a binary value: high responders were given a value of 1, whereas low responders a value of 0. Before the integrative analysis, data was pre-processed using transformation methods available in SIMON knowledge discovery software^31^ centre (mean subtracted) and scale (standard deviation divided) applied before principal component analysis (PCA), t-distributed stochastic neighbour embedding (t-SNE), hierarchical clustering and SIMON analysis, missing values were imputed based on median values (*medianImpute*) (PCA, t-SNE and hierarchical clustering), features with zero-variance (*zv*) and near-zero-variance (*nzv*) were removed (PCA and SIMON), and finally, highly correlated features with cut-off 0.85 (*corr*) were also removed for the supervised machine learning (ML) analysis using SIMON.

#### High-dimensional analysis using t-SNE and clustering analysis

The t-distributed stochastic neighbour embedding (t-SNE) followed by clustering was performed to analyse the pre-processed integrated dataset using SIMON software^31^. Disease severity and timepoint were used as grouping variables, and thus, were excluded from the analysis. T-SNE analysis was performed with 2000 iterations, a perplexity of 30, and a theta of 0.5. Resulting t-SNE maps were used for cluster analysis using model-based clustering algorithm (*mclust*) with seed number 1337 and 3 clusters allowed^72^. To visualize variation of clinical and immunological features across the t-SNE embedding space, we performed hierarchical clustering on t-SNE maps using Euclidean distance, agglomerative hierarchical clustering with Ward and tightest cluster was ordered first.

#### Principal component analysis

Principal component analysis (PCA) was performed on multivariate immunological parameters (continuous variables) with pre-filtering to remove all categorical variables and features with less than 10% of unique values, *i.e*., any column that has number of unique values less than 10% of total number of observations. Disease severity was used as a grouping variable. Quality of variable representations (cos2), variable correlations and contributions (expressed as percentage) of top 10 variables to first two principal components (PCs) were calculated. The correlation between variables and PCs was used as the coordinates of the variables on the PCs. The observations were represented by their projections, while the variables were represented by their correlations^73^.

#### Correlation analysis

Pairwise correlations of immunological parameters on all analysed samples were calculated and visualized as a correlogram using a SIMON software. Spearman’s rank correlation coefficient was computed and indicated on the correlogram by the heat scale. The significance test of correlation coefficients was performed, and values shown on the correlogram were adjusted for multiple testing using false discovery rate (FDR) correction using the Benjamini-Hochberg (B-H) correction at the significance threshold FDR < 0.05. Following correlation analysis, correlogram map was fed into agglomerative hierarchical clustering with Ward algorithm and three major clusters were identified.

#### Hierarchical clustering

Agglomerative hierarchical clustering was performed on the samples with immunological parameters analysed on day 28 pso and visualized as the dendrogram on heatmap using a SIMON software. Cluster analysis was performed using a set of dissimilarities for the number of samples being clustered. Each sample was assigned to its own cluster, then the algorithm iteratively joined the two most similar clusters, continuing until there was just a single cluster. First, the dissimilarity values were computed (dissimilarity matrix calculated using Euclidean method, these values were then fed into hierarchical clustering using complete linkage agglomeration method and finally, dendrogram was plotted (tightest cluster ordered first).

#### Integrative analysis using SIMON

To identify early immunological signature at day 28 pso that can predict if the individual will be high or low responder 6 months pso, we performed SIMON (Sequential Iterative Modeling Over Nigh) analysis^30,31^ Joint predictive analysis on all immunological parameters at day 28 pso (excluding clinical data) was performed using 172 ML algorithms. The outcome was seropositivity status determined 6 months pso. Initial data was split into train/test partition (75%/25%) preserving the balanced distribution of the outcome class (seed number 1337). The dataset had 29% missing values, and missing values were removed using multi-set interaction function (mulset, SIMON software) and 30 resamples were used for the SIMON analysis. The models were evaluated using 10-fold cross-validation on the training sets, and additionally to prevent overfitting on the held-out test sets. The best performing model was built using the Sparse Partial Least Squares (sPLS) algorithm (train AUROC: 0.95 (CI 0.5-1) and test AUROC: 1). In the final step, SIMON calculated the contribution of each feature to the model as variable importance score (scaled to maximum value of 100).

### Statistics & Reproducibility

Statistical analysis was performed using R (https://www.r-project.org/) package ggpubr version 0.4.0, while integrative and machine learning analysis was performed using SIMON software version 0.2.1 (https://genular.org)^30,31^, figures were made with R using R package ggplot2 version 3.3.3^74^, ComplexHeatmap version 2.4.3 and GraphPad Prism 8. Kruskal-Wallis test—unless otherwise specified—was used for comparison of the disease severity groups and high/low responders at different timepoints (with Dunn’s multiple comparison test). Wilcoxon rank-sum test—unless otherwise specified—was employed to compare between study time points. A generalised additive mixed model (GAMM) by restricted maximum likelihood (REML) was used to fit the immunological measures (log10 transformed) using Gaussian process smooth term (R package *gamm4* version 0.2.6^75^). For ICS cytokine expression analyses, data was prepared using PESTEL v2.0 for formatting and baseline subtraction, followed by export of data to SPICE v6.0 for analysis. Spot forming units were enumerated using AID ELISpot 8.0 software on the AID ELR08IFL reader. Statistical significance was set at *P* < 0.05.

Sample size calculation was not performed, all health care workers who had tested SARS-CoV-2 PCR test and were consented to participate in the study were recruited as feasibility allowed. 9 hospitalised patients with severe disease which were included for comparative analysis. All recruited subjects were included, and exclusion criteria wasn’t established prior to testing. Subjects were excluded on a per assay basis where sample availability limited testing or samples failed quality controls. ELISpot—assays where the background was 50 SFU/10e6 or below were accepted as valid; T cell proliferation assay - responses above 1% were considered true positive; ADMP assay—samples were excluded from further analysis if the replicates showed a coefficient of variation of over 25%. The experiments were not randomized, and the investigators were not blinded to allocation during experiments and outcome assessment.

### Reporting summary

Further information on experimental design is available in the Nature Research Reporting Summary linked to this paper.

## Supplementary information

Supplementary Information

Description of Additional Supplementary Files

Supplementary Data 1

Reporting summary

## Data Availability

The processed and integrated data generated in this study have been deposited in the Zenodo data repository [https://zenodo.org/record/4905965].
