# Supplementary material for: Divergent trajectories of antiviral memory after SARS-CoV-2 infection
Source: Nat Commun. 2022 Mar 10;13:1251. doi: 10.1038/s41467-022-28898-1 (PMC8913789; doi:10.1038/s41467-022-28898-1)
Supplement: Supplementary file 1 — Supplementary Information [file 41467_2022_28898_MOESM1_ESM.pdf]

Divergent trajectories of antiviral memory after SARS-CoV-2 infection
Tomic et al

<u>Table of Contents</u>

Supplementary Table 1. Summary table of demographic information for all participants by disease phenotype (Page 2)

Supplementary Table 2. Summary table of descriptive statistics for humoral assays (Pages 3-8)

Supplementary Table 3. Summary statistics for ex vivo interferon-gamma ELISpot assay (Page 9)

Supplementary Table 4. Summary of tables for proliferation assay based on disease phenotype (Pages 10-11)

Supplementary Table 5. Summary of tables for proliferation assay based on peptide pool tested (Page 12)

Supplementary Table 6. List of peptide sequences used in T cell assays (Pages 13-19)

Supplementary Figure 1. Comparison of humoral immune responses in individuals with PCR confirmed SARS-CoV-2 infection (Page 20)

Supplementary Figure 2. Further characterisation of longitudinal humoral immune responses to SARS-CoV-2 and non-SARS-CoV-2 in individuals with PCR confirmed SARS-CoV-2 infection (Page 21)

Supplementary Figure 3. Analysis of T cell responses by clinical disease status, and representative gating strategies (Pages 22-23)

Supplementary Figure 4. Longitudinal ICS analysis of SARS-CoV-2 T cell responses (Page 24)

Supplementary Figure 5. Polyfunctional T cell responses for NP and NSP3B pools (Page 25)

Supplementary Table 1. Summary table of demographic information for all participants by disease phenotype. Number of participants in each cohort – Asymptomatic (asymp), Mild and Severe (requiring hospitalisation and oxygen) are shown, alongside the percentage by sex, age and ethnicity.

		Asymptomatic		Mild		Severe		Total	
**Number enrolled**		**12**	**% of asymp**	**66**	**% of mild**	**7**	**% of severe**	**85**	**% of total**
**Sex**	**Female**	11	92%	48	73%	2	29%	61	72%
	**Male**	1	8%	18	27%	5	71%	24	28%
**Age range (years of age at recruitment)**	**20-29**	2	17%	21	32%	0	0%	23	27%
	**30-39**	4	33%	17	26%	0	0%	21	25%
	**40-49**	2	17%	14	21%	2	29%	18	21%
	**50-59**	3	25%	14	21%	4	57%	21	25%
	**60-69**	1	8%	0	0%	1	14%	2	2%
**Ethnicity**	**Asian**	1	8%	10	15%	1	14%	12	14%
	**Black**	1	8%	0	0%	1	14%	2	2%
	**White**	10	83%	48	73%	3	43%	61	72%
	**Other**	0	0%	4	6%	1	14%	5	6%
	**Unknown**	0	0%	4	6%	1	14%	5	6%

Supplementary Table 2. Summary table of descriptive statistics for humoral assays by disease phenotype. Median with interquartile values across all humoral assays performed (highlighted in grey) on the samples from asymptomatic, mild and sever groups analysed across 6 timepoints as indicated in the column 'Day'. The number of analysed individuals is given in the column 'n'. ND; not-determined.

Day	anti-SARS-CoV-2 nucleocapsid tIgG ELISA (ELISA AU)					
	Asymptomatic		Mild		Severe	
	n	median (IQR)	n	median (IQR)	n	median (IQR)
<20	7	3.40 (2.17-4.31)	39	3.64 (1.09-5.84)	0	ND
28	11	2.15 (1.61-3.81)	60	5.58 (3.83-6.5)	0	ND
56	9	2.45 (1.24-3.44)	63	5.16 (3.09-6.21)	0	ND
90	9	1.46 (0.80-2.24)	51	4.39 (2.41-5.68)	0	ND
120	9	1.22 (0.55-1.51)	51	3.39 (1.50-4.91)	0	ND
180	12	0.84 (0.33-1.12)	64	2.03 (0.72-3.66)	0	ND
Day	anti-SARS-CoV-2 spike tIgG ELISA (ELISA AU)					
	Asymptomatic		Mild		Severe	
	n	median (IQR)	n	median (IQR)	n	median (IQR)
<20	9	341.8 (241.0-897.1)	42	122.4 (61.5-754.8)	8	1182.0 (387.7-1947.0)
28	11	427.2 (260.1-753.0)	64	504.1 (196.6-1361.0)	1	2404.0 (2404.0-2404.0)
56	9	424.4 (287.4-788.3)	62	677.9 (236.1-1532.0)	1	1837.0 (1837.0-1837.0)
90	10	229.8 (114.4-404.9)	51	525.4 (178.5-1006.0)	0	ND
120	10	221.0 (115.9-290.2)	53	365.3 (192.9-765.3)	0	ND
180	12	257.7 (134.7-400.4)	65	399.4 (180.5-710.1)	8	1401.0 (903.6-1616.0)
Day	Pseudoneutralising antibodies (IC50)					
	Asymptomatic		Mild		Severe	
	n	median (IQR)	n	median (IQR)	n	median (IQR)
<20	9	226.20 (118.90-701.60)	40	1261.00 (447.90-3813.00)	8	4923.00 (2135.00-30032.00)
28	11	126.10 (53.86-206.00)	64	817.50 (345.00-2954.00)	0	ND
56	8	127.70 (83.88-242.70)	63	441.8 (212.90-913.00)	1	1346.00 (1346.00-1346.00)
90	10	142.60 (80.18-294.00)	51	393.20 (170.20-747.60)	0	ND
120	10	98.96 (71.32-174.50)	51	243.40 (130.80-430.40)	0	ND
180	12	89.76 (52.01-148.70)	65	182.50 (99.29-479.40)	8	765.1 (457.00-1619.00)
Day	anti SARS-CoV-2-spike IgG memory B cell ELISPOT (ASCs per million PBMCs)					
	Asymptomatic		Mild		Severe	
	n	median (IQR)	n	median (IQR)	n	median (IQR)
<20	7	176.70 (108.30-207.50)	10	25.80 (5.85-49.15)	8	49.00 (26.50-131.80)
28	9	231.70 (180.00-477.40)	44	112.50 (30.43-246.30)	0	ND
56	8	145.90 (34.18-260.00)	35	186.70 (126.70-293.30)	0	ND
90	8	196.70 (153.30-211.40)	31	166.70 (95.00-270.00)	0	ND
120	6	227.70 (145.50-311.10)	30	119.00 (77.50-289.00)	0	ND
180	12	134.2 (95.00-173.70)	47	130.00 (58.30-230.00)	8	649.00 (236.00-1111.00)
Day	anti-SARS-CoV-2 spike IgA memory B cell ELISPOT (ASCs per million PBMCs)					
	Asymptomatic		Mild		Severe	
	n	median (IQR)	n	median (IQR)	n	median (IQR)
<20	7	2.50 (0.00-5.00)	10	0.00 (0.00-10.40)	8	5.00 (2.25-56.50)
28	9	0.00 (0.00-7.50)	44	0.00 (0.00-5.00)	0	ND
56	8	0.00 (0.00-5.03)	35	0.00 (0.00-5.00)	0	ND
90	8	0.00 (0.00-0.00)	31	0.00 (0.00-0.00)	0	ND
120	6	0.00 (0.00-8.75)	30	0.00 (0.00-1.25)	0	ND

180	12	0.00 (0.00-1.46)	47	0.00 (0.00-5.00)	8	0.00 (0.00-12.93)
Day	**IgG1 (ELISA AU)**					
	Asymptomatic		Mild		Severe	
	n	median (IQR)	n	median (IQR)	n	median (IQR)
<20	9	101.50 (18.00-777.50)	10	18.99 (18.00-97.31)	8	1500.00 (1193.00-5106.00)
28	11	18.00 (18.00-612.80)	20	226.9 (54.58-834.30)	1	6835.00 (6835.00-6835.00)
56	9	74.19 (18.00-357.80)	19	338.5 (34.73-826.90)	1	3984.00 (3984.00-3984.00)
90	10	92.07 (18.00-295.90)	15	284.7 (78.74-765.10)	0	ND
120	10	126.40 (18.00-499.60)	15	286 (34.51-818.90)	0	ND
180	12	154.60 (18.00-803.90)	20	459.5 (30.58-941.40)	8	2047.00 (1309.00-3482.00)
Day	**IgG2 (OD units)**					
	Asymptomatic		Mild		Severe	
	n	median (IQR)	n	median (IQR)	n	median (IQR)
<20	9	0.123 (0.118-0.245)	10	0.1145 (0.097-0.1728)	8	0.123 (0.098-0.1485)
28	11	0.099 (0.094-0.125)	20	0.1088 (0.099-0.1449)	1	0.148
56	9	0.097 (0.0905-0.1008)	19	0.117 (0.093-0.15)	1	0.155
90	10	0.097 (0.0935-0.1083)	15	0.1 (0.092-0.14)	0	ND
120	10	0.0985 (0.08725-0.1118)	15	0.112 (0.102-0.148)	0	ND
180	12	0.1015 (0.09175-0.1075)	20	0.109 (0.099-0.1295)	8	0.1255 (0.1058-0.1738)
Day	**IgG3 (ELISA AU)**					
	Asymptomatic		Mild		Severe	
	n	median (IQR)	n	median (IQR)	n	median (IQR)
<20	9	38.08 (27.73-64.09)	10	24.47 (8.00-250.8)	8	254.1 (93.7-553.4)
28	11	18.7 (10.16-49.76)	20	41.99 (16.8-760.4)	1	379.5
56	9	22.76 (10.56-28.34)	19	33.91 (10.59-443.2)	1	605.4
90	10	12.89 (8.00-37.95)	15	32.12 (14.97-256.7)	0	ND
120	10	13.74 (8.00-31.57)	15	29.88 (14.67-217.1)	0	ND
180	12	9.068 (8.00-13.79)	20	17.81 (8.000-65.97)	8	50.39 (19.9-109)
Day	**IgG4 (OD units)**					
	Asymptomatic		Mild		Severe	
	n	median (IQR)	n	median (IQR)	n	median (IQR)
<20	9	0.0605 (0.058-0.064)	10	0.06275 (0.058-0.08113)	8	0.082 (0.08075-0.9225)
28	11	0.08 (0.063-0.0835)	20	0.0815 (0.06088-0.08713)	1	0.0795
56	9	0.0845 (0.08225-0.08575)	19	0.08550 (0.0825-0.087)	1	0.0825
90	10	0.08325 (0.08013-0.08888)	15	0.08550 (0.082-0.0895)	0	ND
120	10	0.083 (0.08038-0.09)	15	0.084 (0.0815-0.0885)	0	ND
180	12	0.08125 (0.078-0.086)	20	0.084 (0.07963-0.08888)	8	0.8725 (0.082-0.093)
Day	**ADNKA (% CD107a expressing NK cells)**					
	Asymptomatic		Mild		Severe	
	n	median (IQR)	n	median (IQR)	n	median (IQR)
<20	9	8.475 (4.188-14.49)	6	13.15 (1.331-19.08)	6	23.91 (20.46-28.12)
28	10	10.76 (3.761-19.68)	17	11.76 (2.77-21.4)	0	ND
56	7	22.54 (12.69-31.59)	18	19.03 (10.2-22.1)	0	ND
90	9	15.25 (7.17-22.02)	13	21.39 (10.62-32.07)	0	ND
120	10	22.62 (14.5-24.4)	15	16.29 (13.79-18.03)	0	ND
180	12	18.54 (8.1-21.44)	20	15.51 (8.6-19.09)	8	23.41 (21.56-25.88)

Day	ADNP (phagocytic score)					
	Asymptomatic		Mild		Severe	
	n	median (IQR)	n	median (IQR)	n	median (IQR)
<20	0	ND	1	0.7071	7	0.2603 (0.1618-0.6023)
28	6	0.2612 (-0.02452-0.41)	12	0.5654 (0.1024-1.154)	1	0.7829
56	9	0.2087 (0.03429-0.487)	17	0.5863 (0.07072-0.9166)	1	0.8277
90	9	0.1695 (-0.01632-0.4668)	13	0.1808 (-0.006721-0.9186)	0	ND
120	8	0.1283 (0.02477-0.3941)	13	0.416 (0.2192-1.087)	0	ND
180	12	0.2889 (0.06631-0.6443)	18	0.685 (0.2207-1.073)	8	0.9643 (0.7522-1.110)

Day	ADMP (phagocytic score)					
	Asymptomatic		Mild		Severe	
	n	median (IQR)	n	median (IQR)	n	median (IQR)
<20	0	ND	1	1.104	4	0.8948 (0.4953-1.231)
28	6	0.2983 (0.04294-0.5566)	13	0.5197 (0.1747-1.087)	1	0.7361
56	9	0.3268 (0.1715-0.5015)	18	0.5135 (0.1884-0.8254)	1	1.06
90	9	0.2479 (0.07806-0.6466)	13	0.4937 (0.2748-0.9776)	0	ND
120	8	0.2539 (0.1134-0.4688)	13	0.4637 (0.2604-0.8632)	0	ND
180	11	0.2683 (0.06291-0.4265)	18	0.5556 (0.2570-0.8358)	4	0.8732 (0.6285-0.9976)

Day	ADCD (complement arbitrary units)					
	Asymptomatic		Mild		Severe	
	n	median (IQR)	n	median (IQR)	n	median (IQR)
<20	9	70.71 (26.23-83.65)	8	44.08 (14.62-706.5)	7	521.1 (496.7-702.8)
28	10	29.58 (23.18-63.61)	19	78.25 949.60-581.1)	1	601.5
56	9	28.83 (16.79-66.81)	19	143.3 (38.75-408.9)	1	459.7
90	9	20.88 (12.92-53.86)	14	75.03 (53.85-225.2)	0	ND
120	10	19.63 (12.38-33.38)	14	84.5 (47.04-148.6)	0	ND
180	10	25.76 (17.16-50.46)	18	65.62 (39.92-131.7)	8	92.56 (68.07-213.7)

Day	MSD-CoV-2-N (MSD units)					
	Asymptomatic		Mild		Severe	
	n	median (IQR)	n	median (IQR)	n	median (IQR)
<20	6	23874 (10566-63490)	12	22008 (2160-288100)	8	351347 (56110-909112)
28	11	9125 (5550-26640)	59	48980 (11134-150139)	1	759067
56	7	14656 (6139-28209)	22	79304 (25109-122795)	1	1060397
90	9	18875 (6235-24250)	15	66980 (21635-100886)	0	ND
120	10	9668 (4468-18295)	15	41029 (17680-63060)	0	ND
180	11	1450 (800-4315)	65	15270 (4603-34135)	8	66976 (28763-130158)

Day	MSD-CoV-2-S (MSD units)					
	Asymptomatic		Mild		Severe	
	n	median (IQR)	n	median (IQR)	n	median (IQR)
<20	6	17508 (13239-35111)	12	2603 (1121-119940)	8	101430 (50745-151061)
28	11	9775 (4675-15650)	59	15934 (6625-58090)	1	172859
56	7	11971 (8063-25715)	22	49465 (12132-103915	1	194617
90	9	10325 (8005-19413)	15	35620 (9060-98421)	0	ND
120	10	11226 (8216-17903)	15	27767 (13731-63222)	0	ND
180	11	5865 (2385-9245)	65	14610 (7848-27165)	8	76010 (50080-83777)

Day	MSD-CoV-2-RBD (MSD units)					
	Asymptomatic		Mild		Severe	
	n	median (IQR)	n	median (IQR)	n	median (IQR)
<20	6	5162 (3514-13439)	12	1137 (428.5-42463)	8	28094 (10097-40119)
28	11	2640 (1825-5985)	59	6389 (2260-16863)	1	62130
56	7	4724 (2667-8375)	22	16408 (2052-38674)	1	73667
90	9	4110 (2913-5609)	15	21405 (2225-27426)	0	ND
120	10	4292 (2454-5672)	15	15729 (3969-24675)	0	ND
180	11	1280 (575-3015)	65	5470 (2618-11740)8	8	27437 (19479-35138)

Day	MSD-CoV-1-S (MSD units)					
	Asymptomatic		Mild		Severe	
	n	median (IQR)	n	median (IQR)	n	median (IQR)
<20	6	2751 (1765-4642)	12	1469 (558.3-8282)	8	11247 (7049-45153)
28	11	1420 (1140-2640)	59	4827 (930-16094)	1	13838
56	7	2049 (1277-5780)	22	6841 (3024-15560)	1	20930
90	9	1710 (1458-4164)	15	7225(2365-19446)	0	ND
120	10	2338 (1670-3455)	15	5045 (3104-9766)	0	ND
180	11	1840 (435-4870)	65	3895 (1908-7018)	8	15296 (7806-34528)

Day	MSD-MERS-S (MSD units)					
	Asymptomatic		Mild		Severe	
	n	median (IQR)	n	median (IQR)	n	median (IQR)
<20	6	540.2 (222.1-8731)	12	1325 (291.2-2613)	8	12607 (6286-24051)
28	11	505(260-1790)	59	1485 (326.2-9838)	1	68698
56	7	741.8 (212.1-5195)	22	2738 (735.7-11182)	1	79785
90	9	565 (282.5-2355)	15	3115 (655-8415)	0	ND
120	10	856.2 (353.2-1256)	15	1875 (650-3528)	0	ND
180	11	856.3 (353.2-1256)	65	1300 (335-5815)	8	3635 (1612-7713)

Day	MSD-229E-S (MSD units)					
	Asymptomatic		Mild		Severe	
	n	median (IQR)	n	median (IQR)	n	median (IQR)
<20	6	24102 (13468-48192)	12	41196 (11351-59135)	8	33216 (7191-51864)
28	11	13690 (5670-22120)	59	27464 (11980-46933)	1	45764
56	7	26430 (14338-35753)	22	39582 (26185-71234)	1	76423
90	9	18135 (10473-37915)	15	45493 (34515-74800)	0	ND
120	10	28051 (11973-46341)	15	44060 (33901-73586)	0	ND
180	11	9600 (5190-18660)	65	28410 (17525-47678)	8	37402 (17322-70656)

Day	MSD-HKU1-S (MSD units)					
	Asymptomatic		Mild		Severe	
	n	median (IQR)	n	median (IQR)	n	median (IQR)
<20	6	16511 (11681-36256)	12	30221 (6930-50917)	8	60514 (27302-92141)
28	11	10980 (6140-19060)	59	33253 (16048-65480)	1	85830
56	7	13990 (8453-47818)	22	32100 (23770-51565)	1	133025
90	9	12306 (9380-34408)	15	24057 (15220-52627)	0	ND
120	10	11651 (7043-30534)	15	24057 (15220-52627)	0	ND
180	11	6490 (3770-18410)	65	16960 (10873-28140)	8	42123 (14326-62614)

Day	MSD-NL63-S (MSD units)					
	Asymptomatic		Mild		Severe	
	n	median (IQR)	n	median (IQR)	n	median (IQR)
<20	6	5522 (4311-9005)	12	3331 (1781-11318)	8	5252 (2413-10385)
28	11	5505 (2305-7765)	59	3855 (1760-9555)	1	10914
56	7	7673 (6180-11166)	22	7109 (2895-11623)	1	19765
90	9	5759 (5283-11355)	15	8040 (3510-15480)	0	ND
120	10	7819 (3625-11602)	15	8096 (3790-11090)	0	ND
180	11	3780 (2600-5445)	65	4590 (2595-9140)	8	5614 (4137-7214)

Day	MSD-OC43-S (MSD units)					
	Asymptomatic		Mild		Severe	
	n	median (IQR)	n	median (IQR)	n	median (IQR)
<20	6	49070 (38844-181033)	12	61023 (26744-131983)	8	168319 (94347-364668)
28	11	44025 (28820-81555)	59	99657 (30725-176897)	1	377370
56	7	48304 (41330-82764)	22	82329 (51781-152439)	1	506545
90	9	54585 (35190-79453)	15	79865 (48805-189773)	0	ND
120	10	44648 (32552-80433)	15	78449 (61302-87133)	0	ND
180	11	29570 (21835-60550)	65	69440 (39608-87133)	8	118990

Day	anti-229E spike IgG memory B cell ELISPOT (ASCs per million PBMCs)					
	Asymptomatic		Mild		Severe	
	n	median (IQR)	n	median (IQR)	n	median (IQR)
<20	7	5 (1.7-6.67)	10	5 (0-11.25)	6	6 (2.25-8.5)
28	2	14.17 (3.33-25)	10	10 (1.693-15)	0	ND
56	0	ND	0	ND	0	ND
90	0	ND	0	ND	0	ND
120	0	ND	0	ND	0	ND
180	11	2.5 (0-7.5)	36	6.67 (1.678-11.7)	5	1.67 (0.0-5.835)

Day	anti- HKU1 spike IgG memory B cell ELISPOT (ASCs per million PBMCs)					
	Asymptomatic		Mild		Severe	
	n	median (IQR)	n	median (IQR)	n	median (IQR)
<20	7	28.3 (5-67.5)	10	187.7 (93.73-300)	6	97 (33.75-199.3)
28	2	85.85 (46.70-125)	9	105 (55-240)	0	ND
56	0	ND	0	ND	0	ND
90	0	ND	0	ND	0	ND
120	0	ND	0	ND	0	ND
180	11	11 (2.5-31.7)	35	16.7 (3.33-40)	5	16.67 (9.585-65.84)

Day	anti-spike NL63 IgG memory B cell ELISPOT (ASCs per million PBMCs)					
	Asymptomatic		Mild		Severe	
	n	median (IQR)	n	median (IQR)	n	median (IQR)
<20	7	6.7 (5-18.3)	10	19.2 (11.28-40.43)	6	9 (0-18)
28	2	84.15 (10-158.3)	10	12.9 (9.575-41.18)	0	ND
56	0	ND	12	12.9 (2.1-22.48)	0	ND
90	0	ND	0	ND	0	ND
120	0	ND	0	ND	0	ND
180	11	10 (1.7-18.3)	24	5 (0.425-15.03)	5	0.00 (0.00-20.84)

Day	anti-spike OC43 IgG memory B cell ELISPOT (ASCs per million PBMCs)					
	Asymptomatic		Mild		Severe	
	n	median (IQR)	n	median (IQR)	n	median (IQR)
<20	7	43.4 (12.6-98.3)	11	141.7 (83.3-300)	6	141.5(34.5-272.8)
28	2	163.4 971.7-255)	10	160.9 (106.7-313.1)	0	ND
56	0	ND	0	ND	0	ND
90	0	ND	0	ND	0	ND
120	0	ND	0	ND	0	ND
180	11	21.7 (13.3-53.3)	35	26.7 (13.33-51.7)	5	20 (11.67-110.4)

Supplementary Table 3. Summary statistics for *ex vivo* interferon-gamma ELISpot assay by time point. *Ex vivo* IFN-γ ELISpot showing the effector T cell responses to summed SARS-CoV-2 peptide pools spanning spike, accessory and structural proteins (S1, S2, M, NP, ORF 3, ORF8, and NSP3B, summed total of SARS-CoV-2 proteins tested and the CEFT positive control peptides for T cell responses) in 78 individuals 28, 90 and 180 days after SARS-CoV-2 (onset of symptoms for mild cases, PCR positive test for asymptomatic participants). SFC / million PBMCs = spot forming cells per million peripheral blood mononuclear cells, with background (negative control wells) subtracted, and a positive response = final count with background subtracted greater than mean + 2 SD of background. D28, d90 and d180 = days after SARS-CoV-2 diagnosis. Significance of differences between timepoints T1, T2 and T3 calculated by paired two-sided Friedman test with Dunn's multiple comparisons test for the n = 57 participants with assays at all three timepoints available.

	**d28 Median (IQR) N = 70**	**d28 No. +ve (%)**	**d90 Median (IQR) N = 64**	**d90 No. +ve (%)**	**d180 Median (IQR) N = 78**	**d180 No. +ve (%)**	**P value d28 VS d90**	**Sign.**	**P value d28 VS d180**	**Sign.**	**P value d90 VS d180**	**Sign.**
**Total positive pools**	373 (201-842)	67/70 (96%)	240 (124-430)	56/63 (89%)	105 (40-218)	61/77 (79%)	0.0006	***	<0.0001	****	0.0002	***
**Total spike**	180 (71-364)	71/70 (87%)	100 (0-175)	41/63 (65%)	43 (0-103)	46/77 (60%)	0.0016	**	<0.0001	****	0.0262	*
**S1**	70 (37-171)	56/70 (80%)	50 (23-89)	39/63 (62%)	25 (15-60)	32/77. (40%)	0.0001	***	<0.0001	****	0.0508	ns
**S2**	83 (27-182)	53/70 (76%)	46 (28-84)	39/63 (62%)	30 (18-58)	33/77. (43%)	0.0027	**	<0.0001	****	0.1646	ns
**M**	63 (25-160)	47/70 (67%)	33 (15-74)	26/63 (41%)	17 (5-43)	24/77 (31%)	<0.0001	****	<0.0001	****	0.03	*
**NP**	121 (73-250)	62/70 (89%)	56 (33-95)	44/63 (70%)	40 (20-74)	47/77 (61%)	<0.0001	****	<0.0001	****	0.0343	*
**ORF3**	15 (5-38)	22/70 (31%)	8 (0-18)	6/63 (8%)	5 (0-13)	2/76 (3%)	0.0028	**	<0.0001	****	0.8959	ns
**ORF8**	3 (0-15)	6/70 (9%)	3 (0-13)	8/63 (13%)	0 (0-9)	3/76 (4%)	0.7126	ns	0.0368	*	0.5576	ns
**NSP3**	18 (3-48)	23/70 (33%)	8 (0-32)	15/63 (24%)	5 (0-15)	12/76 (16%)	0.0245	*	<0.0001	****	0.2946	ns
**CEFT**	488 (209-920)	65/70 (93%)	326 (128-908)	56/63 (89%)	288 (91-823)	66/77 (86%)	0.2034	ns	<0.0001	****	0.0508	ns

Supplementary Table 4. Summary of tables for proliferation assay based on disease phenotype. Proliferation assay summary table highlighting the total number of patients in each group as well as percentage of patients that responding (% positive >1%) to any of the different SARS-CoV-2 proteins tested. Data is presents within the separate T cell compartments analysed (CD4+ and CD8+ T cells).

		**CD4+ T cells**															
		CD4_ D28	CD4_ D180	CD4_ D28	CD4_ D180	CD4_ D28	CD4_ D180	CD4_ D28	CD4_ D180	CD4_ D28	CD4_ D180	CD4_ D28	CD4_ D180	CD4_ D28	CD4_ D180	CD4_ D28	CD4_ D180
	**Disease Phenotype**	**PHA**	**PHA**	**S1**	**S1**	**S2**	**S2**	**M**	**M**	**NP**	**NP**	**ORF3**	**ORF3**	**ORF8**	**ORF8**	**FECT**	**FECT**
Summary (All)	Numbers tested	56.0	56.0	57.0	57.0	57.0	57.0	57.0	57.0	57.0	57.0	57.0	57.0	56.0	56.0	54.0	54.0
	Number positive (>1%)	56.0	56.0	47.0	46.0	45.0	46.0	44.0	32.0	46.0	42.0	30.0	20.0	16.0	14.0	16.0	15.0
	% Positive (>1%)	100.0	100.0	82.5	80.7	78.9	80.7	77.2	56.1	80.7	73.7	52.6	35.1	28.6	25.0	29.6	27.8
Summary (asymptomatic)	Numbers tested with peptide pool	8.0	8.0	8.0	8.0	8.0	8.0	8.0	8.0	8.0	8.0	8.0	8.0	8.0	8.0	8.0	8.0
	Number positive (>1%)	8.0	8.0	4.0	6.0	3.0	6.0	6.0	4.0	4.0	6.0	1.0	4.0	0.0	3.0	1.0	2.0
	% Positive (>1%)	100.0	100.0	50.0	75.0	37.5	75.0	75.0	50.0	50.0	75.0	12.5	50.0	0.0	37.5	12.5	25.0
Summary (Mild)	Numbers tested with peptide pool	48.0	48.0	49.0	49.0	49.0	49.0	49.0	49.0	49.0	49.0	49.0	49.0	49.0	49.0	49.0	49.0
	Number positive (>1%)	48.0	48.0	43.0	40.0	42.0	40.0	38.0	28.0	42.0	36.0	29.0	16.0	16.0	11.0	15.0	13.0
	% Positive (>1%)	100.0	100.0	87.8	81.6	85.7	81.6	77.6	57.1	85.7	73.5	59.2	32.7	32.7	22.4	30.6	26.5

Supplementary Table 4. Summary of tables for proliferation assay based on disease phenotype (continued).

		CD8+ T cells															
		CD8_ D28	CD8_ D180	CD8_ D28	CD8_ D180	CD8_ D28	CD8_ D180	CD8_ D28	CD8_ D180	CD8_ D28	CD8_ D180	CD8_ D28	CD8_ D180	CD8_ D28	CD8_ D180	CD8_ D28	CD8_ D180
	**Disease Phenotype**	**PHA**	**PHA**	**S1**	**S1**	**S2**	**S2**	**M**	**M**	**NP**	**NP**	**ORF3**	**ORF3**	**ORF8**	**ORF8**	**FECT**	**FECT**
Summary (All)	Numbers tested	56.0	56.0	57.0	57.0	57.0	57.0	57.0	57.0	57.0	57.0	57.0	57.0	56.0	56.0	54.0	54.0
	Number positive (>1%)	56.0	56.0	44.0	29.0	39.0	32.0	37.0	19.0	49.0	36.0	21.0	10.0	5.0	5.0	29.0	24.0
	% Positive (>1%)	100.0	100.0	77.2	50.9	68.4	56.1	64.9	33.3	86.0	63.2	36.8	17.5	8.9	8.9	53.7	44.4
Summary (asymptomatic)	Numbers tested with peptide pool	8.0	8.0	8.0	8.0	8.0	8.0	8.0	8.0	8.0	8.0	8.0	8.0	8.0	8.0	8.0	8.0
	Number positive (>1%)	8.0	8.0	2.0	1.0	1.0	2.0	4.0	3.0	5.0	5.0	0.0	3.0	0.0	1.0	2.0	4.0
	% Positive (>1%)	100.0	100.0	25.0	12.5	12.5	25.0	50.0	37.5	62.5	62.5	0.0	37.5	0.0	12.5	25.0	50.0
Summary (Mild)	Numbers tested with peptide pool	48.0	48.0	49.0	49.0	49.0	49.0	49.0	49.0	49.0	49.0	49.0	49.0	49.0	49.0	49.0	49.0
	Number positive (>1%)	48.0	48.0	42.0	28.0	38.0	30.0	33.0	16.0	44.0	31.0	21.0	7.0	5.0	4.0	27.0	20.0
	% Positive (>1%)	100.0	100.0	85.7	57.1	77.6	61.2	67.3	32.7	89.8	63.3	42.9	14.3	10.2	8.2	55.1	40.8

Supplementary Table 5. Summary of tables for proliferation assay based on peptide pool tested. Proliferation assay summary table highlighting median responses with IQR to each of the SARS-CoV-2 peptide pool tested in the asymptomatic and mild disease phenotypes. Data is presents within the separate T cell compartments analysed (CD4+ and CD8+ T cells).

	Median with interquartile range (IQR)							
	CD4				CD8			
	Asymptomatic		Mild		Asymptomatic		Mild	
	Day 28	Day 180	Day 28	Day 180	Day 28	Day 180	Day 28	Day 180
**M (%)**	2.7 (1.9 - 9.5)	7.6 (1.9 - 20.5)	13.7 (8.4 - 29.6)	7.9 (3.9 - 18.2)	2.7 (1.5 - 3.0)	2.3 (1 - 4)	3.4 (1.6 - 7.9)	2.6 (1.6 - 5.5)
**NP (%)**	4.2 (2.6 - 16.5)	11.6 (2.9 - 20.1)	12.5 (6.8 - 22.7)	6.7 (2.6 - 21.7)	5.3 (3.7 - 13.5)	5.1 (3.3 - 21.3)	10.2 (5.9 - 22.6)	4.5 (1.8 - 12.1)
**ORF3 (%)**	1.9 (1.9 - 1.9)	1.9 (1.4 - 4.2)	3.5 (2.5 - 7)	2.9 (1.8 - 5.6)	0	1.9 (1.2 - 2.3)	3.8 (1.7 - 9.2)	4.3 (2 - 7.14)
**ORF8 (%)**	0	2.6 (2.2 - 2.9)	3.1 (1.6 - 6.4)	2.3 (1.9 - 3.1)	0	3.8 (3.8 - 3.8)	3.7 (2.7 - 13.6)	1.5 (1.1 - 2.0)
**Total responses to at least peptide outside spike**	87.50%	75%	86%	75.00%	62.50%	75%	91%	71%

Supplementary Table 6. List of peptide sequences used for T cell assays. Peptide pools covering NSP3B, ORF3, M, ORF8, NP, S1 and S2 SARS-CoV-2 proteins, including their peptide IDs and sequences.

Peptide Pool (NSP3B)	Peptide ID	Peptide Sequence
NSP3B	ORF1a/1ab_207	DWSYSGQSTQLGIEFLKR
	ORF1a/1ab_208	TQLGIEFLKRGDKSVYY
	ORF1a/1ab_209	LKRGDKSVYYTSNPTTF
	ORF1a/1ab_210	VYYTSNPTTFHLDGEVI
	ORF1a/1ab_211	TTFHLDGEVITFDNLKTL
	ORF1a/1ab_212	VITFDNLKTLLSLREVR
	ORF1a/1ab_213	KTLLSLREVRTIKVFTTV
	ORF1a/1ab_214	VRTIKVFTTVDNINL
	ORF1a/1ab_215	VFTTVDNINLHTQVVDM
	ORF1a/1ab_216	INLHTQVVDMSMTYGQQF
	ORF1a/1ab_217	DMSMTYGQQFGPTYL
	ORF1a/1ab_218	YGQQFGPTYLDGADVTKI
	ORF1a/1ab_219	YLDGADVTKIKPHNSHEG
	ORF1a/1ab_220	KIKPHNSHEGKTFYVL
	ORF1a/1ab_221	SHEGKTFYVLPNDDTLRV
	ORF1a/1ab_222	VLPNDDTLRVEAFEYY
	ORF1a/1ab_223	TLRVEAFEYYHTTDPSFL
	ORF1a/1ab_224	YYHTTDPSFLGRYMSAL
	ORF1a/1ab_225	SFLGRYMSALNHTKKWKY
	ORF1a/1ab_226	ALNHTKKWKYPQVNGL
	ORF1a/1ab_227	KWKYPQVNGLTSIKW
	ORF1a/1ab_228	QVNGLTSIKWADNNCYL
	ORF1a/1ab_229	IKWADNNCYLATALLTL
	ORF1a/1ab_230	CYLATALLTLQQIELKF
	ORF1a/1ab_231	LTLQQIELKFNPPAL
	ORF1a/1ab_232	IELKFNPPALQDAYYRAR
	ORF1a/1ab_233	ALQDAYYRARAGEAANF
	ORF1a/1ab_234	RARAGEAANFCALILAY
	ORF1a/1ab_235	ANFCALILAYCNKTVGEL
	ORF1a/1ab_236	AYCNKTVGELGDVRETM
	ORF1a/1ab_237	GELGDVRETMSYLFQHAN
	ORF1a/1ab_238	TMSYLFQHANLDSCKRVL
	ORF1a/1ab_239	ANLDSCKRVLNVVCK
	ORF1a/1ab_240	CKRVLNVVCKTCGQQQTT
	ORF1a/1ab_241	CKTCGQQQTTLKGVEAVM
	ORF1a/1ab_242	TTLKGVEAVMYMGTLSY
	ORF1a/1ab_243	AVMYMGTLSYEQFKKGV
	ORF1a/1ab_244	LSYEQFKKGVQIPCTCGK
	ORF1a/1ab_245	GVQIPCTCGKQATKYLV
	ORF1a/1ab_246	CGKQATKYLVQQESPFVM
	ORF1a/1ab_247	LVQQESPFVMMSAPPAQY
	ORF1a/1ab_248	VMMSAPPAQYELKHGTF
	ORF1a/1ab_249	AQYELKHGTFTCASEY
	ORF1a/1ab_250	HGTFTCASEYTGNYQCGH
	ORF1a/1ab_251	EYTGNYQCGHYKHITSK
	ORF1a/1ab_252	CGHYKHITSKETLYCI
	ORF1a/1ab_253	ITSKETLYCIDGALLTK
	ORF1a/1ab_254	YCIDGALLTKSSEYKGPI
	ORF1a/1ab_255	TKSSEYKGPITDVFYK
	ORF1a/1ab_256	KGPITDVFYKENSYTTTI
	ORF1a/1ab_257	YKENSYTTTIKPVTYKL
	ORF1a/1ab_258	TTIKPVTYKLDGVVCTEI
	ORF1a/1ab_259	KLDGVVCTEIDPKLDNYY
	ORF1a/1ab_260	EIDPKLDNYYKKDNSYF
	ORF1a/1ab_261	NYYKKDNSYFTEQPIDLV
	ORF1a/1ab_262	YFTEQPIDLVPNQPY
	ORF1a/1ab_263	PIDLVPNQPYPNASFDNF
	ORF1a/1ab_264	PYPNASFDNFKFVCDNIK
	ORF1a/1ab_265	NFKFVCDNIKFADDLNQL
	ORF1a/1ab_266	IKFADDLNQLTGYKK
	ORF1a/1ab_267	DLNQLTGYKKPASRELKV

Peptide Pool (NSP3B)	Peptide ID	Peptide Sequence
	ORF1a/1ab_268	KKPASRELKVTFFPDL
	ORF1a/1ab_269	ELKVTFFPDLNGDVVAI
	ORF1a/1ab_270	PDLNGDVVAIDYKHY
	ORF1a/1ab_271	DVVAIDYKHYTPSFKK
	ORF1a/1ab_272	YKHYTPSFKKGAKLLHK
	ORF1a/1ab_273	FKKGAKLLHKPIVWHV
	ORF1a/1ab_274	LLHKPIVWHVNNATNK
	ORF1a/1ab_275	VWHVNNATNKATYKPNTW
	ORF1a/1ab_276	NKATYKPNTWCIRCLW
	ORF1a/1ab_277	PNTWCIRCLWSTKPV
	ORF1a/1ab_278	IRCLWSTKPVETSNSFDV
	ORF1a/1ab_279	PVETSNSFDVLKSEDAQG
	ORF1a/1ab_280	DVLKSEDAQGMDNLACED
	ORF1a/1ab_281	QGMDNLACEDLKPVSEEV
	ORF1a/1ab_282	EDLKPVSEEVVENPTIQK
	ORF1a/1ab_283	EVVENPTIQKDVLECNVK
	ORF1a/1ab_284	QKDVLECNVKTTEVVGDI
	ORF1a/1ab_285	VKTTEVVGDIILKPANNS
	ORF1a/1ab_286	DIILKPANNSLKITEEV
	ORF1a/1ab_287	NNSLKITEEVGHTDLM
	ORF1a/1ab_288	TEEVGHTDLMAAYVDNSS
	ORF1a/1ab_289	LMAAYVDNSSLTIKK
	ORF1a/1ab_290	VDNSSLTIKKPNELSRVL
	ORF1a/1ab_291	KKPNELSRVLGLKTL
	ORF1a/1ab_292	LSRVLGLKTLATHGLAAV
	ORF1a/1ab_293	TLATHGLAAVNSVPWDTI
	ORF1a/1ab_294	AVNSVPWDTIANYAKPFL
	ORF1a/1ab_295	TIANYAKPFLNKVVSTTT
	ORF1a/1ab_296	FLNKVVSTTTNIVTRCL
	ORF1a/1ab_297	TTTNIVTRCLNRVCTNYM
	ORF1a/1ab_298	CLNRVCTNYMPYFFTLLL
	ORF1a/1ab_299	YMPYFFTLLLQLCTFTR
	ORF1a/1ab_300	LLLQLCTFTRSTNSRIK
	ORF1a/1ab_301	FTRSTNSRIKASMPTTI
	ORF1a/1ab_302	RIKASMPTTIAKNTVKSV
	ORF1a/1ab_303	TIAKNTVKSVGKFCL
	ORF1a/1ab_304	TVKSVGKFCLEASFNYLK
	ORF1a/1ab_305	CLEASFNYLKSPNFSKLI
	ORF1a/1ab_306	LKSPNFSKLINIIIWFLL

Peptide Pool (ORF3)	Peptide ID	Peptide Sequence
ORF3	ORF3a_1	MDLFMRIFTIGTVTLK
	ORF3a_2	IFTIGTVTLKQGEIK
	ORF3a_3	TVTLKQGEIKDATPSDFV
	ORF3a_4	IKDATPSDFVRATATIPI
	ORF3a_5	FVRATATIPIQASLPFGW
	ORF3a_6	PIQASLPFGWLIVGVALL
	ORF3a_7	GWLIVGVALLAVFQSASK
	ORF3a_8	LLAVFQSASKIITLKKRW
	ORF3a_9	SKIITLKKRWQLALSKGV
	ORF3a_10	RWQLALSKGVHFVCNLLL
	ORF3a_11	GVHFVCNLLLLFVTVY
	ORF3a_12	NLLLLFVTVYSHLLLV
	ORF3a_13	VTVYSHLLLVAAGLEAPF
	ORF3a_14	LVAAGLEAPFLYLYALVY
	ORF3a_15	PFLYLYALVYFLQSINFV
	ORF3a_16	VYFLQSINFVRIIMRLWL
	ORF3a_17	FVRIIMRLWLCWKCRSK
	ORF3a_18	LWLCWKCRSKNPLLY
	ORF3a_19	KCRSKNPLLYDANYFLCW
	ORF3a_20	LYDANYFLCWHTNCYDY
	ORF3a_21	LCWHTNCYDYCIPYNSV
	ORF3a_22	YDYCIPYNSVTSSIVI
	ORF3a_23	YNSVTSSIVITSGDGTTS
	ORF3a_24	VITSGDGTTSPISEHDY

Peptide Pool (ORF3)	Peptide ID	Peptide Sequence
	ORF3a_25	TTSPISEHDYQIGGYTEK
	ORF3a_26	DYQIGGYTEKWESGVK
	ORF3a_27	YTEKWESGVKDCVVLHSY
	ORF3a_28	VKDCVVLHSYFTSDYYQL
	ORF3a_29	SYFTSDYYQLYSTQL
	ORF3a_30	DYYQLYSTQLSTDTGV
	ORF3a_31	STQLSTDTGVEHVTFFIY
	ORF3a_32	GVEHVTFFIYNKIVDEPE
	ORF3a_33	IYNKIVDEPEEHVQIHTI
	ORF3a_34	PEEHVQIHTIDGSSGVV
	ORF3a_35	HTIDGSSGVVNPVMEPIY
	ORF3a_36	VVNPVMEPIYDEPTTTTS
	ORF3a_37	PVMEPIYDEPTTTTSVPL

Peptide Pool (M)	Peptide ID	Peptide Sequence
M	M(ORF5)_1	MADSNGTITVEELKKLL
	M(ORF5)_2	ITVEELKKLLEQWNLVI
	M(ORF5)_3	KLLEQWNLVIGFLFLTWI
	M(ORF5)_4	VIGFLFLTWICLLQFAY
	M(ORF5)_5	TWICLLQFAYANRNRFLY
	M(ORF5)_6	AYANRNRFLYIIKLIFLW
	M(ORF5)_7	LYIIKLIFLWLLWPVTL
	M(ORF5)_8	FLWLLWPVTLACFVLAAV
	M(ORF5)_9	TLACFVLAAVYRINWI
	M(ORF5)_10	LAAVYRINWITGGIAIAM
	M(ORF5)_11	WITGGIAIAMACLVGLMW
	M(ORF5)_12	AMACLVGLMWLSYFIASF
	M(ORF5)_13	MWLSYFIASFRLFARTR
	M(ORF5)_14	ASFRLFARTRSMWSF
	M(ORF5)_15	FARTRSMWSFNPETNILL
	M(ORF5)_16	SFNPETNILLNVPLHGTI
	M(ORF5)_17	LLNVPLHGTILTRPLL
	M(ORF5)_18	HGTILTRPLLESELVI
	M(ORF5)_19	RPLLESELVIGAVILR
	M(ORF5)_20	ELVIGAVILRGHLRI
	M(ORF5)_21	AVILRGHLRIAGHHLGR
	M(ORF5)_22	LRIAGHHLGRCDIKDLPK
	M(ORF5)_23	GRCDIKDLPKEITVATSR
	M(ORF5)_24	PKEITVATSRTLSYYKL
	M(ORF5)_25	TSRTLSYYKLGASQRV
	M(ORF5)_26	YYKLGASQRVAGDSGF
	M(ORF5)_27	SQRVAGDSGFAAYSRYRI
	M(ORF5)_28	GFAAYSRYRIGNYKL
	M(ORF5)_29	SRYRIGNYKLNTDHSSSS
	M(ORF5)_30	KLNTDHSSSSDNIALLV
	M(ORF5)_31	KLNTDHSSSSDNIALLVQ

Peptide Pool (ORF8)	Peptide ID	Peptide Sequence
ORF8	ORF8_1	MKFLVFLGIITTVAAF
	ORF8_2	LGIITTVAAFHQECSL
	ORF8_3	VAAFHQECSLQSCTQHQP
	ORF8_4	SLQSCTQHQPYVVDDPCP
	ORF8_5	QPYVVDDPCPIHFYSKWY
	ORF8_6	CPIHFYSKWYIRVGARK
	ORF8_7	KWYIRVGARKSAPLIEL
	ORF8_8	ARKSAPLIELCVDEAGSK
	ORF8_9	ELCVDEAGSKSPIQYIDI
	ORF8_10	SKSPIQYIDIGNYTVSCL
	ORF8_11	DIGNYTVSCLPFTINCQE
	ORF8_12	CLPFTINCQEPKLGSLVV
	ORF8_13	QEPKLGSLVVRCSFYEDF
	ORF8_14	VVRCSFYEDFLEYHDVRV
	ORF8_15	FYEDFLEYHDVRVVLDFI

Peptide Pool (NP)	Peptide ID	Peptide Sequence
NP	N(ORF9)_1	MSDNGPQNQRNAPRITF
	N(ORF9)_2	NQRNAPRITFGGPSDSTG
	N(ORF9)_3	TFGGPSDSTGSNQNGER
	N(ORF9)_4	STGSNQNGERSGARSKQR
	N(ORF9)_5	ERSGARSKQRRPQGL
	N(ORF9)_6	RSKQRRPQGLPNNTASWF
	N(ORF9)_7	GLPNNTASWFTALTQHGK
	N(ORF9)_8	WFTALTQHGKEDLKFPR
	N(ORF9)_9	HGKEDLKFPRGQGVPI
	N(ORF9)_10	KFPRGQGVPINTNSSPDD
	N(ORF9)_11	PINTNSSPDDQIGYYRR
	N(ORF9)_12	PDDQIGYYRRATRRIR
	N(ORF9)_13	YYRRATRRIRGGDGKMK
	N(ORF9)_14	RIRGGDGKMKDLSPRWYF
	N(ORF9)_15	MKDLSPRWYFYYLGTGPE
	N(ORF9)_16	YFYYLGTGPEAGLPY
	N(ORF9)_17	GTGPEAGLPYGANKDGII
	N(ORF9)_18	PYGANKDGIIWVATEGAL
	N(ORF9)_19	IIWVATEGALNTPKDHI
	N(ORF9)_20	GALNTPKDHIGTRNPANN
	N(ORF9)_21	HIGTRNPANNAAIVLQL
	N(ORF9)_22	ANNAAIVLQLPQGTTLPK
	N(ORF9)_23	QLPQGTTLPKGFYAEGSR
	N(ORF9)_24	PKGFYAEGSRGGSQASSR
	N(ORF9)_25	SRGGSQASSRSSSRSR
	N(ORF9)_26	ASSRSSSRSRNSSRNSTP
	N(ORF9)_27	SRNSSRNSTPGSSRGTSP
	N(ORF9)_28	TPGSSRGTSPARMAGNGG
	N(ORF9)_29	SPARMAGNGGDAALALLL
	N(ORF9)_30	GGDAALALLLLDRLNQL
	N(ORF9)_31	LLLLDRLNQLESKMSGK
	N(ORF9)_32	NQLESKMSGKGQQQQGQT
	N(ORF9)_33	GKGQQQQGQTVTKKSAAE
	N(ORF9)_34	QTVTKKSAAEASKKPRQK
	N(ORF9)_35	AEASKKPRQKRTATKAY
	N(ORF9)_36	RQKRTATKAYNVTQAFGR
	N(ORF9)_37	AYNVTQAFGRRGPEQTQG
	N(ORF9)_38	GRRGPEQTQGNFGDQELI
	N(ORF9)_39	QGNFGDQELIRQGTDYK
	N(ORF9)_40	ELIRQGTDYKHWPQIAQF
	N(ORF9)_41	YKHWPQIAQFAPSASAFF
	N(ORF9)_42	QFAPSASAFFGMSRIGM
	N(ORF9)_43	AFFGMSRIGMEVTPSGTW
	N(ORF9)_44	GMEVTPSGTWLTYTGAIK
	N(ORF9)_45	TWLTYTGAIKLDDKDPNF
	N(ORF9)_46	IKLDDKDPNFKDQVILL
	N(ORF9)_47	PNFKDQVILLNKHIDAYK
	N(ORF9)_48	LLNKHIDAYKTFPPTEPK
	N(ORF9)_49	YKTFPPTEPKKDKKKK
	N(ORF9)_50	TEPKKDKKKKADETQAL
	N(ORF9)_51	KKKADETQALPQRQKK
	N(ORF9)_52	TQALPQRQKKQQTVTLL
	N(ORF9)_53	QKKQQTVTLLPAADLDDF
	N(ORF9)_54	LLPAADLDDFSKQLQQSM
	N(ORF9)_55	DFSKQLQQSMSSADSTQA

Peptide Pool (S1)	Peptide ID	Peptide Sequence
	S_1	MFVFLVLLPLVSSQCVNL
	S_2	PLVSSQCVNLTTRTQL
	S_3	CVNLTTRTQLPPAYTNSF
	S_4	QLPPAYTNSFTRGVYY
	S_5	TNSFTRGVYYPDKVFR
	S_6	GVYYPDKVFRSSVLHSTQ
	S_7	FRSSVLHSTQDLFLPFF
	S_8	STQDLFLPFFSNVTWF

Peptide Pool (S1)	Peptide ID	Peptide Sequence
S1	S_9	LPFFSNVTWFHAIHV
	S_10	NVTWFHAIHVSGTNGTKR
	S_11	HVSGTNGTKRFDNPVLPF
	S_12	KRFDNPVLPFNDGVYF
	S_13	VLPFNDGVYFASTEKSNI
	S_14	YFASTEKSNIIRGWIF
	S_15	KSNIIRGWIFGTTLDSK
	S_16	WIFGTTLDSKTQSLLIV
	S_17	DSKTQSLLIVNNATNVVI
	S_18	IVNNATNVVIKVCEFQF
	S_19	VVIKVCEFQFCNDPFLGV
	S_20	QFCNDPFLGVYYHKNNK
	S_21	LGVYYHKNNKSWMESEFR
	S_22	NKSWMESEFRVYSSANNC
	S_23	FRVYSSANNCTFEYV
	S_24	SANNCTFEYVSQPFLMDL
	S_25	YVSQPFLMDLEGKQGNFK
	S_26	DLEGKQGNFKNLREFVFK
	S_27	FKNLREFVFKNIDGYFKI
	S_28	FKNIDGYFKIYSKHTPI
	S_29	FKIYSKHTPINLVRDL
	S_30	HTPINLVRDLPQGFSAL
	S_31	RDLPQGFSALEPLVDLPI
	S_32	ALEPLVDLPIGINITRF
	S_33	LPIGINITRFQTLLALHR
	S_34	RFQTLLALHRSYLTPGDS
	S_35	HRSYLTPGDSSSGWTAGA
	S_36	DSSSGWTAGAAAYYVGYL
	S_37	GAAAYYVGYLQPRTFLLK
	S_38	YLQPRTFLLKYNENGTI
	S_39	LLKYNENGTITDAVDCAL
	S_40	TITDAVDCALDPLSETK
	S_41	CALDPLSETKCTLKSFTV
	S_42	TKCTLKSFTVEKGIY
	S_43	KSFTVEKGIYQTSNFRV
	S_44	GIYQTSNFRVQPTESIVR
	S_45	RVQPTESIVRFPNITNL
	S_46	IVRFPNITNLCPFGEVF
	S_47	TNLCPFGEVFNATRFASV
	S_48	VFNATRFASVYAWNRKRI
	S_49	SVYAWNRKRISNCVADY
	S_50	KRISNCVADYSVLYNSAS
	S_51	DYSVLYNSASFSTFKCY
	S_52	SASFSTFKCYGVSPTKL
	S_53	KCYGVSPTKLNDLCFTNV
	S_54	KLNDLCFTNVYADSFVIR
	S_55	NVYADSFVIRGDEVRQI
	S_56	VIRGDEVRQIAPGQTGKI
	S_57	QIAPGQTGKIADYNYKL
	S_58	GKIADYNYKLPDDFTGCV
	S_59	KLPDDFTGCVIAWNSNNL
	S_60	CVIAWNSNNLDSKVGGNY
	S_61	NLDSKVGGNYNYLYRLFR
	S_62	NYNYLYRLFRKSNLKPF
	S_63	LFRKSNLKPFERDISTEI
	S_64	PFERDISTEIYQAGSTPC
	S_65	EIYQAGSTPCNGVEGF
	S_66	STPCNGVEGFNCYFPL
	S_67	VEGFNCYFPLQSYGF
	S_68	CYFPLQSYGFQPTNGVGY
	S_69	GFQPTNGVGYQPYRVVVL
	S_70	GYQPYRVVVLSFELL
	S_71	RVVVLSFELLHAPATV
	S_72	FELLHAPATVCGPKK
	S_73	APATVCGPKKSTNLVKNK
	S_74	KKSTNLVKNKCVNFNF

Peptide Pool (S1)	Peptide ID	Peptide Sequence
	S_75	VKNKCVNFNFNGLTGTGV
	S_76	NFNGLTGTGVLTESNKKF
	S_77	GVLTESNKKFLPFQQFGR
	S_78	KFLPFQQFGRDIADTTDA
	S_79	GRDIADTTDAVRDPQTL
	S_80	TDAVRDPQTLEILDI
	S_81	DPQTLEILDITPCSFGGV
	S_82	DITPCSFGGVSVITPGTN
	S_83	GVSVITPGTNTSNQVAVL
	S_84	TNTSNQVAVLYQDVNCTE
	S_85	VLYQDVNCTEVPVAI
	S_86	VNCTEVPVAIHADQL
	S_87	VPVAIHADQLTPTWRVY
	S_88	DQLTPTWRVYSTGSNVF
	S_89	RVYSTGSNVFQTRAGCLI
	S_90	VFQTRAGCLIGAEHV
	S_91	AGCLIGAEHVNNSYECDI
	S_92	HVNNSYECDIPIGAGI
	S_93	ECDIPIGAGICASYQTQT

Peptide Pool (S2)	Peptide ID	Peptide Sequence
S2	S_94	GICASYQTQTNSPRRAR
	S_95	TQTNSPRRARSVASQSII
	S_96	ARSVASQSIIAYTMSL
	S_97	QSIIAYTMSLGAENSVAY
	S_98	SLGAENSVAYSNNSIAI
	S_99	VAYSNNSIAIPTNFTISV
	S_100	AIPTNFTISVTTEILPV
	S_101	ISVTTEILPVSMTKTSV
	S_102	LPVSMTKTSVDCTMYI
	S_103	KTSVDCTMYICGDSTECS
	S_104	YICGDSTECSNLLLQY
	S_105	TECSNLLLQYGSFCTQL
	S_106	LQYGSFCTQLNRALTGI
	S_107	TQLNRALTGIAVEQDK
	S_108	LTGIAVEQDKNTQEVF
	S_109	EQDKNTQEVFAQVKQIYK
	S_110	VFAQVKQIYKTPPIKDF
	S_111	IYKTPPIKDFGGFNFSQI
	S_112	DFGGFNFSQILPDPSK
	S_113	FSQILPDPSKPSKRSFI
	S_114	PSKPSKRSFIEDLLFNKV
	S_115	FIEDLLFNKVTLADAGFI
	S_116	KVTLADAGFIKQYGDCL
	S_117	GFIKQYGDCLGDIAARDL
	S_118	CLGDIAARDLICAQKF
	S_119	ARDLICAQKFNGLTVL
	S_120	AQKFNGLTVLPPLLTDEM
	S_121	VLPPLLTDEMIAQYTSAL
	S_122	EMIAQYTSALLAGTI
	S_123	YTSALLAGTITSGWTF
	S_124	AGTITSGWTFGAGAALQI
	S_125	TFGAGAALQIPFAMQMAY
	S_126	QIPFAMQMAYRFNGIGV
	S_127	MAYRFNGIGVTQNVLY
	S_128	GIGVTQNVLYENQKLI
	S_129	NVLYENQKLIANQFNSAI
	S_130	LIANQFNSAIGKIQDSL
	S_131	SAIGKIQDSLSSTASAL
	S_132	DSLSSTASALGKLQDVV
	S_133	SALGKLQDVVNQNAQAL
	S_134	DVVNQNAQALNTLVKQL
	S_135	QALNTLVKQLSSNFGAI

Peptide Pool (S2)	Peptide ID	Peptide Sequence
	S_136	KQLSSNFGAISSVLNDIL
	S_137	AISSVLNDILSRLDKV
	S_138	NDILSRLDKVEAEVQIDR
	S_139	KVEAEVQIDRLITGRL
	S_140	QIDRLITGRLQSLQTYV
	S_141	GRLQSLQTYVTQQLIR
	S_142	QTYVTQQLIRAAEIR
	S_143	QQLIRAAEIRASANL
	S_144	AAEIRASANLAATKM
	S_145	ASANLAATKMSECVL
	S_146	AATKMSECVLGQSKRVDF
	S_147	VLGQSKRVDFCGKGYHLM
	S_148	DFCGKGYHLMSFPQSAPH
	S_149	LMSFPQSAPHGVVFLHV
	S_150	APHGVVFLHVTYVPAQEK
	S_151	HVTYVPAQEKNFTTAPAI
	S_152	EKNFTTAPAICHDGKAHF
	S_153	AICHDGKAHFPREGVFV
	S_154	AHFPREGVFVSNGTHWFV
	S_155	FVSNGTHWFVTQRNFY
	S_156	HWFVTQRNFYEPQII
	S_157	QRNFYEPQIITTDNTFV
	S_158	QIITTDNTFVSGNCDVVI
	S_159	FVSGNCDVVIGIVNNTVY
	S_160	VIGIVNNTVYDPLQPEL
	S_161	TVYDPLQPELDSFKEEL
	S_162	PELDSFKEELDKYFK
	S_163	FKEELDKYFKNHTSPDV
	S_164	YFKNHTSPDVDLGDISGI
	S_165	DVDLGDISGINASVVNI
	S_166	SGINASVVNIQKEIDRL
	S_167	VNIQKEIDRLNEVAKNL
	S_168	DRLNEVAKNLNESLIDL
	S_169	KNLNESLIDLQELGKY
	S_170	LIDLQELGKYEQYIKWPW
	S_171	KYEQYIKWPWYIWLGFI
	S_172	WPWYIWLGFIAGLIAIVM
	S_173	FIAGLIAIVMVTIMLCCM
	S_174	VMVTIMLCCMTSCCSCLK
	S_175	CMTSCCSCLKGCCSCGSC
	S_176	LKGCCSCGSCCKFDEDDS
	S_177	SCCKFDEDDSEPVLKGVK
	S_178	FDEDDSEPVLKGVKLHYT

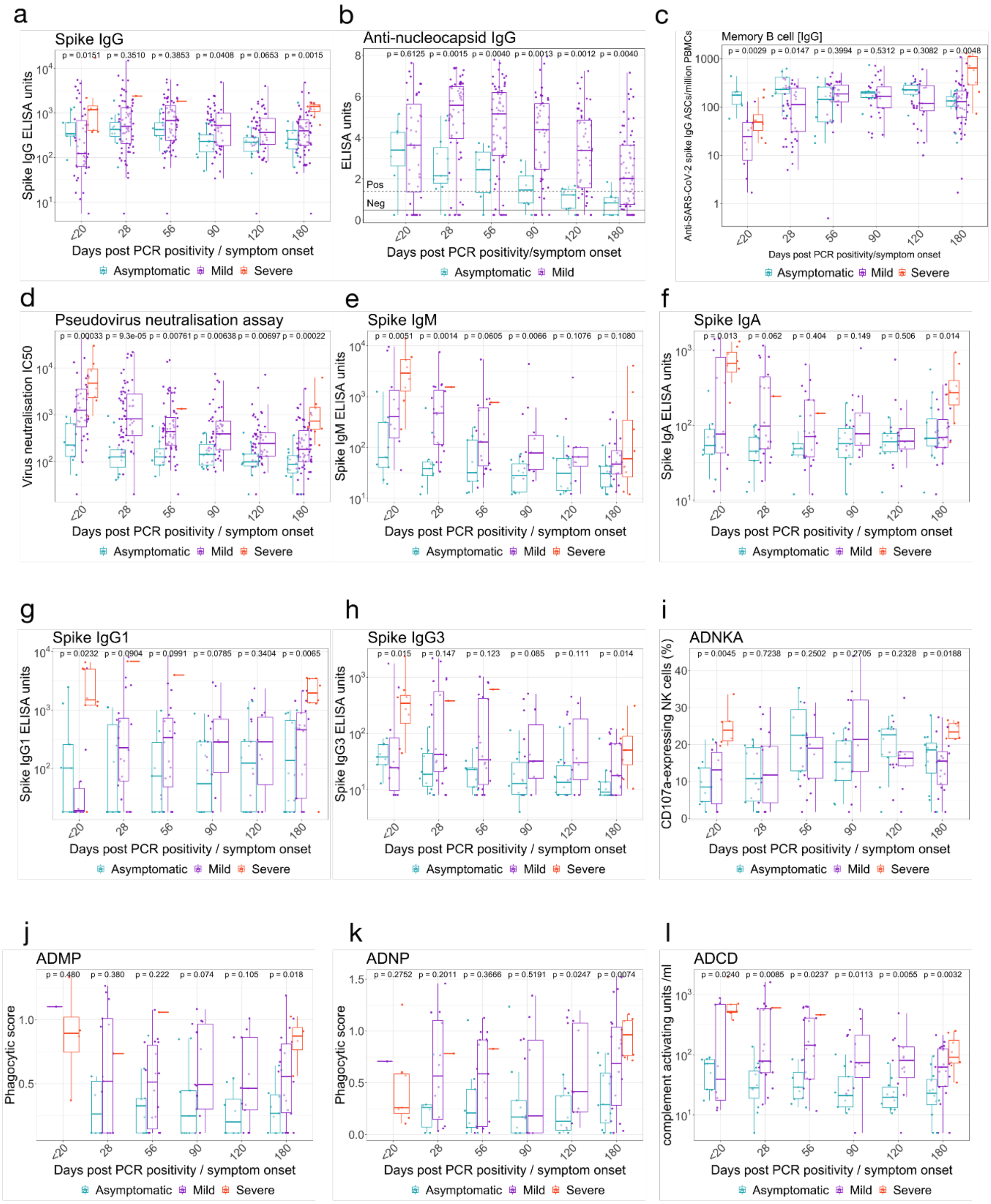

Supplementary Figure 1. Comparison of humoral immune responses in individuals with PCR confirmed SARS-CoV-2 infection. Asymptomatic (blue), mild (purple) or severe (red). Comparison of total IgG specific to the SARS-CoV-2 (a) nucleocapsid and (b) spike glycoprotein, quantification of (c) IgG memory B cells specific to the spike glycoprotein, and (d) pseudoneutralisating antibody titres. Anti-SARS-CoV-2 spike antibody (e-f) isotypes, (g-h) subclasses and (i-l) Fc-mediated effector functions were also compared. The boxplots all display the median values with the first and third quartile, and the whiskers represent the highest and lowest values no more than 1.5 times the interquartile range from the corresponding hinge. Kruskal-Wallis (one-way ANOVA on ranks) test was used for comparison of the disease severity groups. See Table S1 for number of individuals evaluated per assay.

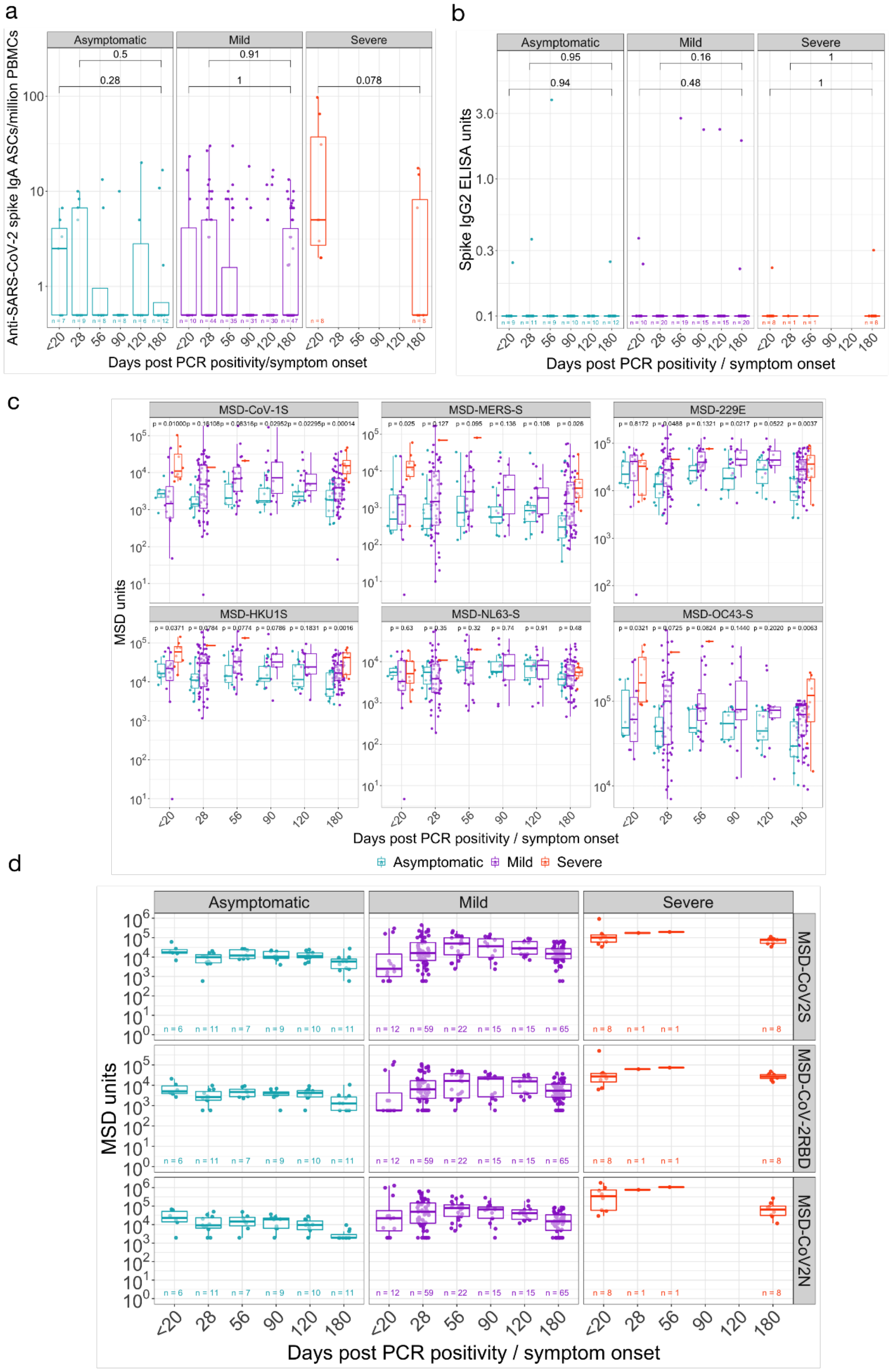

Supplementary Figure 2. Further characterisation of longitudinal humoral immune responses to SARS-CoV-2 and non-SARS-CoV-2 in individuals with PCR confirmed SARS-CoV-2 infection. Asymptomatic (blue), mild (purple) or severe (red). SARS-CoV-2 spike glycoprotein-specific (a) IgA memory B cells and (b) IgG2 subclass antibodies. Meso Scale Discovery (MSD) multiplexed immunoassay platform measurements of antibody levels specific to (c) non-SARS-CoV-2 coronaviruses spike glycoproteins compared across the diseases cohorts at each sampling timepoint, and (d) SARS-CoV-2 spike glycoprotein over time. The boxplots all display the median values with the first and third quartile, and the whiskers represent the highest and lowest values no more than 1.5 times the interquartile range from the corresponding hinge. Kruskal-Wallis (one-way ANOVA on ranks) test was used for comparison of the disease severity groups (c), while Wilcoxon rank-sum two-sided test was employed to compare between study time points (a,b). Number of individuals (n) evaluated per assay indicated.

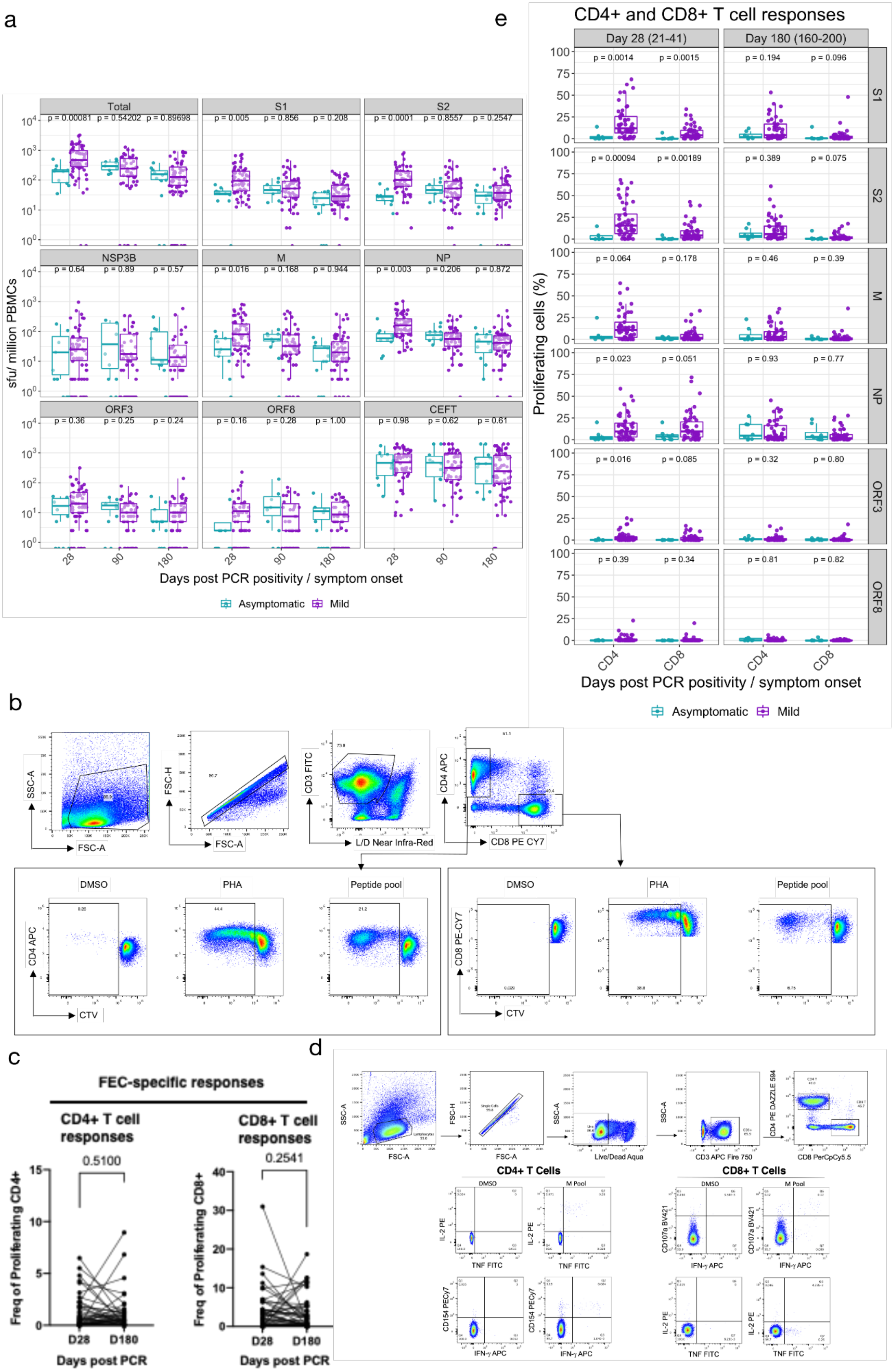

Supplementary Figure 3. Analysis of T cell responses by clinical disease status, and representative gating strategies. (a) Comparison of ex vivo IFN-γ ELISpot responses in healthcare workers post asymptomatic (n=12) vs mild (n=66) SARS-CoV-2 infection. Summed total of SARS-CoV-2 proteins, S1, S2, NSP3B, M, NP, ORF 3, ORF8, and the CEFT positive control peptides for T cell

responses at 28, 90 and 180 days post onset of symptoms for mild cases, PCR positive test for asymptomatic participants. (b) to (d) PBMC were stimulated with 1ug/ml of peptide pools or 2ug/ml of PHA and 0.2% DMSO for proliferation assay (b and c) or 2ug/mL of peptide pool for ICS assay (d). Representative gating strategy is shown in for T cell proliferation assay (b) CEFT control responses in the proliferation assay is shown in (c) and Representative ICS plots are shown in (d) for both CD4+ and CD8+ T cell responses. Comparison by two-tailed Mann-Whitney test. (e) Comparison of proliferative T cell responses to SARS-CoV-2 peptide pools at 1- and 6-months post infection between disease severity groups. The boxplots all display the median values with the first and third quartile, and the whiskers represent the highest and lowest values no more than 1.5 times the interquartile range from the corresponding hinge. Kruskal-Wallis (one-way ANOVA on ranks) test was used for comparison of the disease severity groups in (a) and (e).

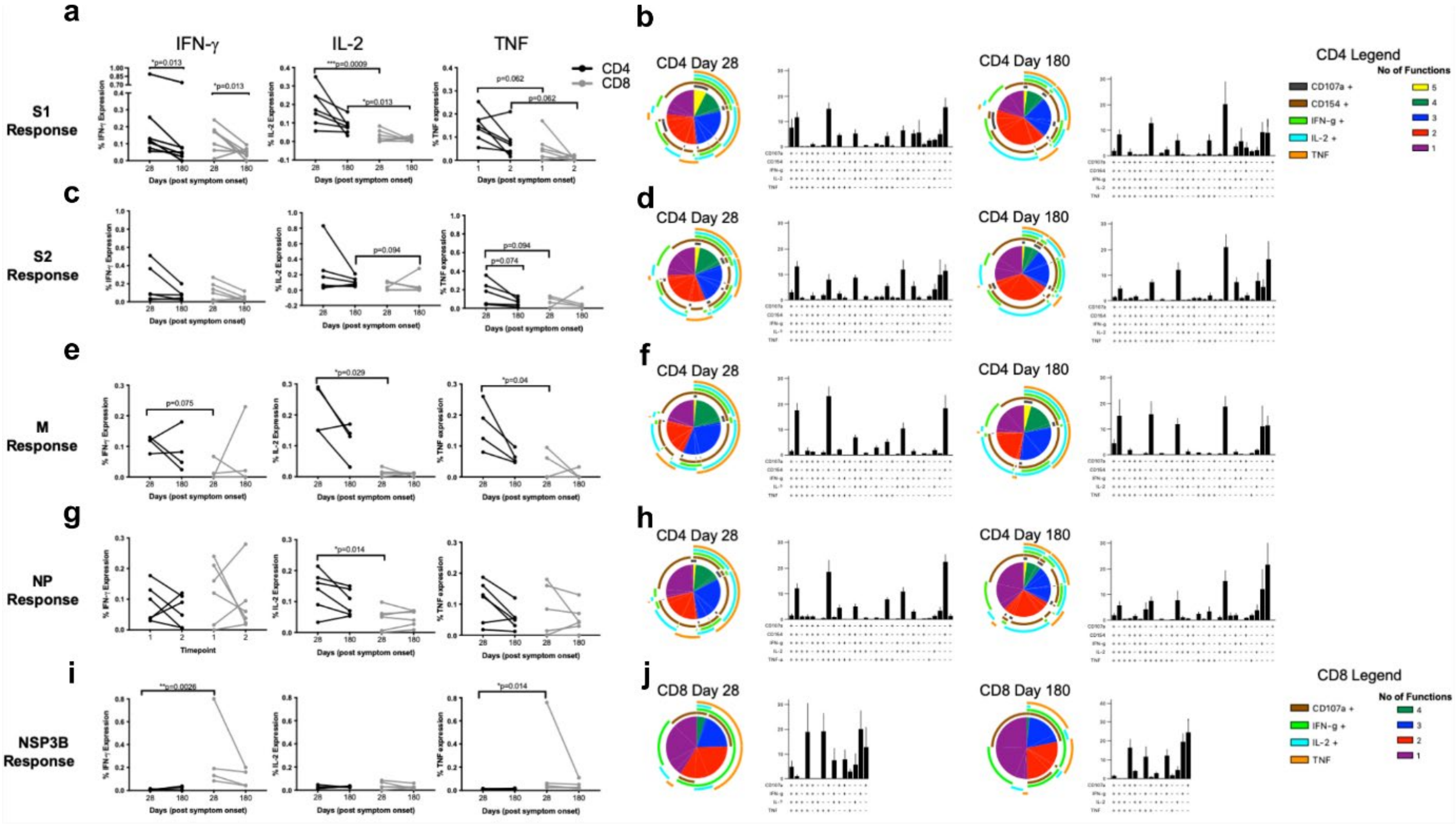

Supplementary Figure 4. Longitudinal ICS analysis of SARS-CoV-2 T cell responses to SARS-CoV-2 pools. Individuals with ELISpot levels >100 spots/10^6 PBMC for a particular peptide pool at timepoint 2 were studied longitudinally at two timepoints using ICS. A total of n=18 individuals were studied with n=15 providing paired analysis. 1^6 frozen PBMC were stimulated with 2ug/mL of the listed peptide along with 1ug/mL CD28 and CD49d antibodies for 6 hours. Expression levels of IFN-γ, IL-2, and TNF in CD4+ T cells (black) and CD8+ T cells (grey) are shown at two timepoints for S1 (a), S2 (c), M (e), NP (g) and NSP3B (i). A paired Friedman test was performed with the two-stage step-up method of Benjamini, Kriegar and Yekutieli correction for multiple comparisons. P values <0.1 are shown. Polyfunctional analysis was performed on both timepoints for individuals with sufficient cell populations for polyfunctional analysis. Data for CD4+ T cells are shown at two timepoints for S1 (b) (n=7, n=8 timepoints 1 and 2 respectively), S2 (d) (n= 5, n=6), M (f) (n=4, n=4), and NP pools (h) (n= 6, n=5). Data for CD8+ T cells are shown at two timepoints for NSP3B pools (j) (n=3, n=5).

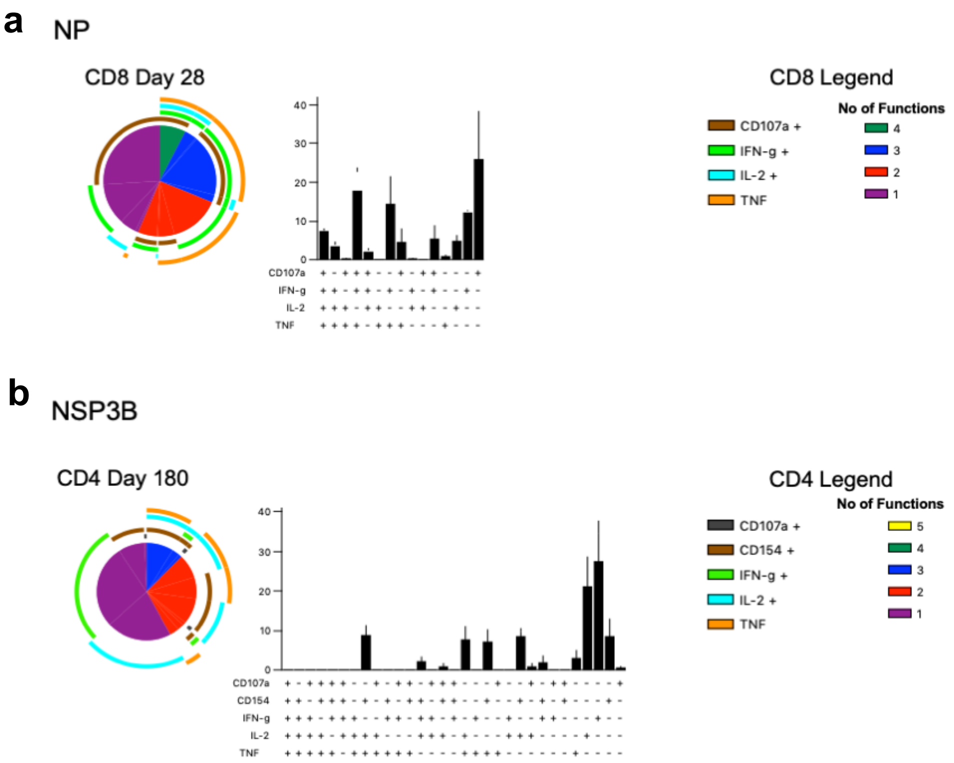

Supplementary Figure 5. Polyfunctional T cell responses for NP and NSP3B pools. Polyfunctional analysis was performed as in Supplemental Figure 4. (a) shows CD8+ T cell polyfunctional analysis for the NP pool at day 28 (n=3). (b) shows CD4+ T cell polyfunctional analysis for the NSP3V pool at day 180 (n=5).
