# Supplementary material for: Divergent trajectories of antiviral memory after SARS-CoV-2 infection
Source: Nat Commun. 2022 Mar 10;13:1251. doi: 10.1038/s41467-022-28898-1 (PMC8913789; doi:10.1038/s41467-022-28898-1)
Supplement: Supplementary file 2 — Description of Additional Supplementary Files [file 41467_2022_28898_MOESM2_ESM.pdf]

Description of Additional Supplementary Files

Supplementary Data 1: Raw data and summary table for proliferation assay
