# Supplementary material for: Divergent trajectories of antiviral memory after SARS-CoV-2 infection
Source: Nat Commun. 2022 Mar 10;13:1251. doi: 10.1038/s41467-022-28898-1 (PMC8913789; doi:10.1038/s41467-022-28898-1)
Supplement: Supplementary file 4 — Reporting summary [file 41467_2022_28898_MOESM4_ESM.pdf]

nature portfolio

Corresponding author(s): Eleanor Barnes and Adriana Tomic

Last updated by author(s): Jan 18, 2022

Reporting Summary

Nature Portfolio wishes to improve the reproducibility of the work that we publish. This form provides structure for consistency and transparency in reporting. For further information on Nature Portfolio policies, see our Editorial Policies and the Editorial Policy Checklist.

Statistics

For all statistical analyses, confirm that the following items are present in the figure legend, table legend, main text, or Methods section.

n/a	Confirmed	
☐	☒	The exact sample size (*n*) for each experimental group/condition, given as a discrete number and unit of measurement
☐	☒	A statement on whether measurements were taken from distinct samples or whether the same sample was measured repeatedly
☐	☒	The statistical test(s) used AND whether they are one- or two-sided<br>*Only common tests should be described solely by name; describe more complex techniques in the Methods section.*
☐	☒	A description of all covariates tested
☐	☒	A description of any assumptions or corrections, such as tests of normality and adjustment for multiple comparisons
☐	☒	A full description of the statistical parameters including central tendency (e.g. means) or other basic estimates (e.g. regression coefficient) AND variation (e.g. standard deviation) or associated estimates of uncertainty (e.g. confidence intervals)
☐	☒	For null hypothesis testing, the test statistic (e.g. *F*, *t*, *r*) with confidence intervals, effect sizes, degrees of freedom and *P* value noted<br>*Give P values as exact values whenever suitable.*
☒	☐	For Bayesian analysis, information on the choice of priors and Markov chain Monte Carlo settings
☐	☒	For hierarchical and complex designs, identification of the appropriate level for tests and full reporting of outcomes
☒	☐	Estimates of effect sizes (e.g. Cohen's *d*, Pearson's *r*), indicating how they were calculated

Our web collection on statistics for biologists contains articles on many of the points above.

Software and code

Policy information about availability of computer code

Data collection	ELISpot plates were read using an AID ELISpot Reader (v.4.0), T cell proliferation assay was performed on a MACSQuant 10 flow cytometer, Intracellular cytokine staining was performed on BD LSRII.
Data analysis	Statistical analysis was performed using R (https://www.r-project.org/) package ggpubr version 0.4.0, integrative analysis was performed using SIMON software version 0.2.1 (https://genular.org), figures were made with R using R package ggplot2 version 3.3.3, ComplexHeatmap version 2.4.3 and GraphPad Prism 8. A generalised additive mixed model (GAMM) by restricted maximum likelihood (REML) was used to fit the immunological measures (log10 transformed) using Gaussian process smooth term (R package gamm4 version 0.2.6). ICS cytokine expression analyses was performed using PESTEL v2.0 and SPICE v6.0. Spot forming units were enumerated using AID ELISpot 8.0 software on the AID ELR08IFL reader.

For manuscripts utilizing custom algorithms or software that are central to the research but not yet described in published literature, software must be made available to editors and reviewers. We strongly encourage code deposition in a community repository (e.g. GitHub). See the Nature Portfolio guidelines for submitting code & software for further information.

Data

Policy information about availability of data

All manuscripts must include a data availability statement. This statement should provide the following information, where applicable:

- Accession codes, unique identifiers, or web links for publicly available datasets
- A description of any restrictions on data availability
- For clinical datasets or third party data, please ensure that the statement adheres to our policy

The processed and integrated data generated in this study have been deposited in the Zenodo data repository [https://zenodo.org/record/4905965].

Field-specific reporting

Please select the one below that is the best fit for your research. If you are not sure, read the appropriate sections before making your selection.

- [x] Life sciences
- [ ] Behavioural & social sciences
- [ ] Ecological, evolutionary & environmental sciences

For a reference copy of the document with all sections, see nature.com/documents/nr-reporting-summary-flat.pdf

Life sciences study design

All studies must disclose on these points even when the disclosure is negative.

Sample size

Sample size calculation was not performed, all health care workers (HCW) who had tested SARS-COV-2 PCR test and were consented to participate in the study were recruited as feasibility allowed. 9 hospitalised patients with severe disease which were included for comparative analysis. N = 78 is a robust sample size for a comprehensive longitudinal study including B and T cell studies compared to the existing literature. For example, Dan et al Science 2021 10.1126/science.abf4063 included N = 43 at 6 months.

A posthoc analysis demonstrated the study had 81.5% power with alpha = 0.05 to detect a difference between the symptomatic and asymptomatic groups.

Data exclusions

All recruited subjects were included and exclusion criteria wasn't established prior to testing. Subjects were excluded on a per assay basis where sample availability limited testing or samples failed quality controls. ELISpot - assays where the background was 50 SFU/10e6 or below were accepted as valid; T cell proliferation assay - responses above 1% were considered true positive; ADMP assay - samples were excluded from further analysis if the replicates showed a coefficient of variation of over 25%.

Replication

All assays reported in the manuscript were well established and had undergone successful replication using pilot studies.Standardised ELISpots assays were run in duplicates. Proliferation assays were validated on HCV Seronegative individuals using HCV specific peptide pools to determine level of background noise from the assay. All antibodies used were of the same clone throughout the duration of the study and different vials (if from different lots) titrated to obtain similar frequencies and MFI. DMSO control with matching percent DMSO was also used in all assays to account for DMSO content in peptide pools.

Randomization

Randomization was not appropriate for this study as samples were selected based on the SARS-CoV-2 PCR test and disease severity.

Blinding

Blinding was not performed as analysis focused on understanding the immune responses in different disease severity. Laboratory staff were blinded to disease severity status (symptomatic v mild) but may not have been blinded to study timepoint due to sample inventory including date and all participants were infected in a narrow timeframe (April-June 2020).

Reporting for specific materials, systems and methods

We require information from authors about some types of materials, experimental systems and methods used in many studies. Here, indicate whether each material, system or method listed is relevant to your study. If you are not sure if a list item applies to your research, read the appropriate section before selecting a response.

Materials & experimental systems

n/a	Involved in the study	
☐	☒	Antibodies
☒	☐	Eukaryotic cell lines
☒	☐	Palaeontology and archaeology
☒	☐	Animals and other organisms
☐	☒	Human research participants
☒	☐	Clinical data
☒	☐	Dual use research of concern

Methods

n/a	Involved in the study	
☒	☐	ChIP-seq
☐	☒	Flow cytometry
☒	☐	MRI-based neuroimaging

Antibodies

Antibodies used

CD3 FITC (Company: Biolegend, Catalogue number: 300446, Clone: UCHT1, Lot number: B227643); CD4 AF700 (Company: BD Bioscience, Catalogue number: 557922, Clone: RPA-T4, Lot number: B291544); CD8 PECY7 (Company: Biolegend, Catalogue number: 301012, Clone: RPA-T8, Lot number: 9009966); CD3 APC Fire 750 (Company: Biolegend, Catalogue Number: 344840, Clone: SK7, Lot Number: B286176); CD4 PE Dazzle 594 (Company: Biolegend, Catalogue Number: 300548, Clone: RPA-T4, Lot Number: 300548); CD8 PerCpCy5.5 (Company: Biolegend, Catalogue Number: 301032, Clone: RPA-T8, Lot Number: B271242); CD107a BV421 (Company: BD Biosciences, Catalogue Number: 562623, Clone: H4A3, Lot Number: 0003034); CD154 PECy7 (Company: Biolegend, Catalogue Number: 310832, Clone: 24-31, Lot Number: B254398); IFN-g APC (Company: BD Biosciences, Catalogue Number: 554702, Clone: B27, Lot Number: 7121607), IL-2 PE (Company: Biolegend, Catalogue Number: 500307, Clone: MQ1-17H12, Lot Number: B272813), TNF-a FITC (Company: BD Biosciences, Catalogue Number: 554512, Clone: Mab11, Lot Number: 8323911).

Validation	All antibodies were titrated upon receipt to validate performance, in addition to the validation performed by manufacturer and provided on their website/quality check summary.

Human research participants

Policy information about studies involving human research participants

Population characteristics	The age, sex and ethnicity are shown in Supplementary Table 1.
Recruitment	Health care workers (HCW) were recruited from Oxford University Hospitals NHS Foundation Trust after a positive SARS-CoV-2 PCR test in April-May 2020, including 66 volunteers with symptomatic disease (fever, shortness of breath, cough, loss of taste or smell, sore throat, coryza or diarrhoea) and 12 asymptomatic HCW who did not report any symptoms of COVID-19 in 2020 prior to staff screening or in the seven days following testing positive. Nine hospitalised patients with severe disease were included for comparative analysis. All subjects were seropositive for anti-spike IgG antibodies by ELISA.<br>Healthcare workers registering for the hospital's PCR testing service (both screening and testing of symptomatic people) had the option to tick a box to indicate their willingness to participate in research. Those who received a positive PCR result who had consented to research participation then received an invitation email from our study team. Those who responded were enrolled in the study. Some subjects volunteered for the study through word of mouth via colleagues participating. Study recruitment bias therefore includes potentially reducing participation by those with less access to email or who chose not to participate in testing. This is a minority of people in the hospital and we do not believe this would impact our findings on the immune response to SARS-CoV-2.
Ethics oversight	Mild and asymptomatic participants were recruited under ethics approved by the research ethics committee (REC) at Yorkshire & The Humber - Sheffield (GI Biobank Study 16/YH/0247). Participants with severe disease were recruited after consenting into either the CMORE study protocol (research ethics committee (REC): Northwest – Preston, REC reference 20/NW/0235) and / or Sepsis Immunomics protocol [Oxford Research Ethics Committee C, reference 19/SC/0296]). The study was conducted according to the principles of the Declaration of Helsinki (2008) and the International Conference on Harmonization (ICH) Good Clinical Practice (GCP) guidelines. Written informed consent was obtained for all participants enrolled in the study.

Note that full information on the approval of the study protocol must also be provided in the manuscript.

Flow Cytometry

Plots

Confirm that:

- [x] The axis labels state the marker and fluorochrome used (e.g. CD4-FITC).
- [x] The axis scales are clearly visible. Include numbers along axes only for bottom left plot of group (a 'group' is an analysis of identical markers).
- [x] All plots are contour plots with outliers or pseudocolor plots.
- [x] A numerical value for number of cells or percentage (with statistics) is provided.

Methodology

Sample preparation	PBMCs were isolated by density gradient centrifugation using LymphoprepTM (p=1.077 g/ml, Stem Cell Technologies), washed twice with RPMI 1640 (Sigma, St. Louis, MO, USA) containing 10% heat-inactivated FCS (Sigma), 1mM Pen/Strep (100U/mL) and 2mM L-glutamine (100 ug/mL) (Sigma) or AutoMACS Rinse Buffer and resuspended in R10 or AutoMACs Rinse Buffer and counted using the Guava® ViaCountTM assay on the Muse Cell Analyzer (Luminex Cooperation). PBMCs were frozen and stored in liquid nitrogen. To obtain plasma, the uppermost fraction following the initial Lymphoprep centrifugation above was collected and centrifuged at 2000g for 10 minutes to remove platelets before storage at -80°C.
Instrument	BD LSR II and Miltenyi MACQuant
Software	Data was prepared using PESTEL v2.0 for formatting and baseline subtraction, followed by export of data to SPICE v6.0 for analysis.
Cell population abundance	No manipulation of samples post PBMC isolation was performed. Assays were setup with fresh or crypreserved total PBMC.
Gating strategy	Lymphocyte gating was performed for the proliferation assay using FCS and SSC, doublets were gated out based on FCS gates, live cells were gated using CD3 and live/dead cell dyes, CD4 and CD8 lineages were gated using CD4 and CD8 markers. Representative gates for the gating strategy are available in the Supplementary figure 3.

- [x] Tick this box to confirm that a figure exemplifying the gating strategy is provided in the Supplementary Information.
